# The Effects of Capillary Transit Time Heterogeneity (CTH) on the Cerebral Uptake of Glucose and Glucose Analogs: Application to FDG and Comparison to Oxygen Uptake

**DOI:** 10.3389/fncom.2016.00103

**Published:** 2016-10-13

**Authors:** Hugo Angleys, Sune N. Jespersen, Leif Østergaard

**Affiliations:** ^1^Center of Functionally Integrative Neuroscience and MINDLab, Aarhus UniversityAarhus, Denmark; ^2^Department of Physics and Astronomy, Aarhus UniversityAarhus, Denmark; ^3^Department of Neuroradiology, Aarhus University HospitalAarhus, Denmark

**Keywords:** aerobic glycolysis, capillary transit time heterogeneity, glucose, fluorodeoxyglucose, lumped constant

## Abstract

Glucose is the brain's principal source of ATP, but the extent to which cerebral glucose consumption (CMR_glc_) is coupled with its oxygen consumption (CMRO_2_) remains unclear. Measurements of the brain's oxygen-glucose index OGI = CMRO_2_/CMR_glc_ suggest that its oxygen uptake largely suffices for oxidative phosphorylation. Nevertheless, during functional activation and in some disease states, brain tissue seemingly produces lactate although cerebral blood flow (CBF) delivers sufficient oxygen, so-called aerobic glycolysis. OGI measurements, in turn, are method-dependent in that estimates based on glucose analog uptake depend on the so-called lumped constant (LC) to arrive at CMR_glc_. Capillary transit time heterogeneity (CTH), which is believed to change during functional activation and in some disease states, affects the extraction efficacy of oxygen from blood. We developed a three-compartment model of glucose extraction to examine whether CTH also affects glucose extraction into brain tissue. We then combined this model with our previous model of oxygen extraction to examine whether differential glucose and oxygen extraction might favor non-oxidative glucose metabolism under certain conditions. Our model predicts that glucose uptake is largely unaffected by changes in its plasma concentration, while changes in CBF and CTH affect glucose and oxygen uptake to different extents. Accordingly, functional hyperemia facilitates glucose uptake more than oxygen uptake, favoring aerobic glycolysis during enhanced energy demands. Applying our model to glucose analogs, we observe that LC depends on physiological state, with a risk of overestimating relative increases in CMR_glc_ during functional activation by as much as 50%.

## Introduction

Normal brain function depends critically on a constant energy supply, and therefore on moment-to-moment regulation of oxygen and glucose availability in brain tissue. In mammals, under normal physiologic conditions, glucose is the major metabolic fuel in the brain (Berg et al., 2012). Glucose can be metabolized through different metabolic pathways: through glycolysis, each glucose molecule is first metabolized into two molecules of pyruvate, with the concomitant production of two molecules of ATP. Then, in the brain, pyruvate can be converted to lactate in the absence of oxygen (anaerobic glycolysis), or completely oxidized to CO_2_ under aerobic conditions, so-called oxidative phosphorylation, generating much more ATP (30 molecules per glucose molecule). In the normal adult brain, studies in many laboratories have established the overall stoichiometry of oxygen and glucose utilization. OGI is approximately equal to 5.5 and is therefore close to the theoretical maximum of 6 which corresponds to the complete oxidation of glucose (Edvinsson and Krause, 2002). This has led to the long held thesis that brain glucose metabolism is mainly oxidative. However, this idea appears to be contradicted by a phenomenon called ‘aerobic glycolysis’ which is the disproportionately higher utilization of glucose than O_2_ in the normoxic working brain. This phenomenon suggests that lactate is produced although oxygen level are seemingly sufficient to support oxidative phosphorylation (Edvinsson and Krause, 2002). Some studies indicate that aerobic glycolysis is linked to functional activation. For example, during sensory stimulation or mental tasks in human, subjects have been reported to evoke 30–50% increases in blood flow and CMR_glc_ with little or no change in CMRO_2_ when measured by PET (Fox et al., 1988) or from arterio-venous metabolite differences (Madsen et al., 1995). Traditionally, lactic acid production is believed to be related to a lack of oxygen. Indeed, it takes place in particular in skeletal muscles when energy needs outpace the ability to transport oxygen and in solid cancer tumors, which are known to grow more rapidly than the blood vessels nourish them, and therefore to experience hypoxia. Under these conditions, glycolysis and subsequent lactic acid fermentation becomes the primary source of ATP (Berg et al., 2012). In the brain, it is unclear whether lactate production occurs in conjunction with local hypoxia. Hypotheses have been formulated to provide a deeper understanding of aerobic glycolysis. For example, the astrocyte-neuron lactate shuttle (ANLS) hypothesis (Pellerin and Magistretti, 2003) proposes that an enhancement of aerobic glycolysis occurs in astrocytes in response to neuronal activation. The ANLS hypothesis predicts a reduction in the molar ratio of oxygen to glucose consumption during activation, but the proposed compartmentalization of glucose metabolism among cell types remains controversial (Chih and Roberts, 2003; Hertz, 2004). Moreover, many aspects of aerobic glycolysis remain poorly understood, including its dependency on stimulus type, duration, and magnitude (Edvinsson and Krause, 2002).

Biophysical models of oxygen delivery (Jespersen and Østergaard, 2012; Angleys et al., 2015; Rasmussen et al., 2015) suggest that the redistribution of blood flow across the capillary bed, as indexed by the extent of capillary transit time heterogeneity (CTH), affects the effective permeability surface area of the capillary bed, and hence the extraction efficacy of freely diffusible molecules. Unlike oxygen, however, glucose does not diffuse freely across the capillary membrane, and the extent to which CTH affects glucose delivery is therefore less clear. Glucose extraction is instead mediated by glucose transporters, namely the glucose transporter GLUT-1 at the blood brain barrier (BBB). GLUT-1 transporters have a maximal rate of operation, and facilitated diffusion of glucose can therefore become saturated in capillaries which support a sufficiently high rate of glucose delivery.

To examine whether the biophysical properties of oxygen and glucose extraction, respectively, introduces a need for brain parenchyma to utilize aerobic glycolysis under certain conditions, we set out to develop a model that infers glucose extraction and consumption from cerebral blood flow (CBF) as well as CTH. We then combine our predictions with those yielded by a comprehensive model of oxygen extraction (Angleys et al., 2015). Using data from *in vivo* rat studies, we predict the extent to which oxygen extraction capacity varies relative to that of glucose, especially among physiological conditions, as characterized by their capillary mean transit time (MTT) and CTH.

To compare our model prediction to *in vivo* measurements, we partly rely on measurements of glucose analogs uptake rather than native glucose. We therefore extend the model to predict FDG uptake, as well as the lumped constant (LC), which, in steady state, equals the ratio of glucose tracer to native glucose extraction and hence allows to relate the concentration of glucose trapped in the tissue to CMR_glc_. Our calculation to determine LC is analog to, but differs from Holden and colleagues' (Holden et al., 1991) in that the effects of CTH are taken into account and glucose transport across the BBB is described differently (see Section Methods). After quantifying the extent to which LC changes between physiological states, we examine whether this re-evaluation of LC can explain the apparent discrepancy between PET and NMRS measurement of CMR_glc_ in the literature. Finally, we discuss possible clinical applications of our model findings.

## Methods

### Assumptions to describe glucose transport across the BBB

In this study, we employ reversible, symmetric Michaelis-Menten kinetics to describe the transport of glucose across the capillary membrane. We treat the endothelium as a single membrane and thus neglect the endothelial compartment. In fact, when employing Michaelis-Menten kinetics, it is possible to show that treating the double membrane as a whole is mathematically almost equivalent to considering two identical membranes, each with identical Michaelis-Menten parameter K_T_ and twice the maximum transport capacity v_max_t_ (exact equivalence is obtained when considering non-reversible Michaelis-Menten). Treating the two membranes as a single barrier is also supported experimentally, for example by the reexamination of non-reversible Michaelis-Menten kinetics across a double-membrane system carried out by Gjedde and Christensen (1984). We discuss the appropriateness of reversible Michaelis-Menten kinetics further below.

### Aim of the model and general procedure

**Figure 1:**
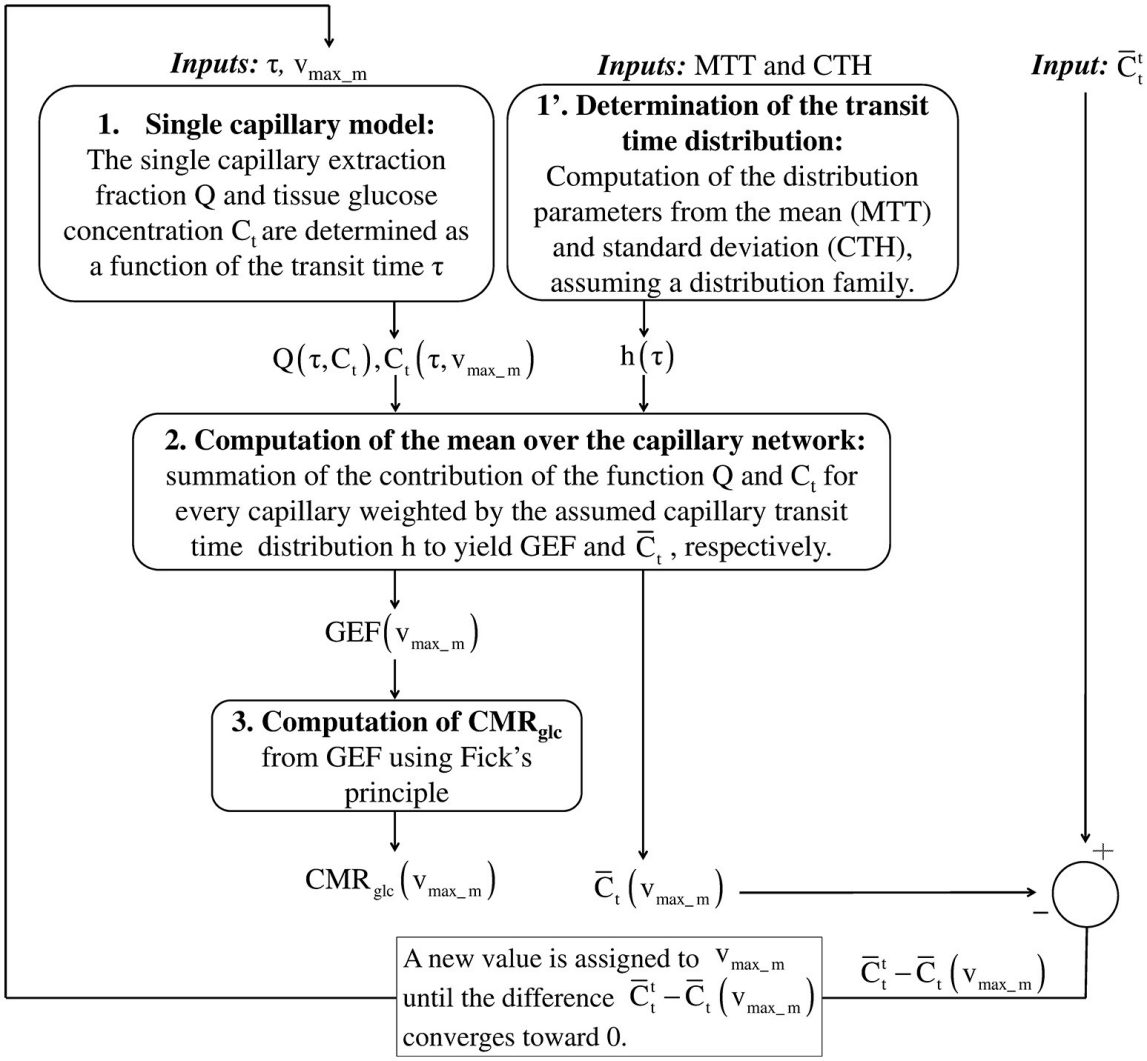
**Schematic illustrating the procedure for computing GEF and CMR_**glc**_, given MTT, CTH and a (target) mean glucose concentration in the tissue ${\bar{\text{C}}}_{\text{t}}^{\text{t}}$**. In the first step, the true value of the effective maximum rate v_max_m_ is not known. Assuming a value for v_max_m_, the mean concentration in the tissue ${\bar{\text{C}}}_{\text{t}}$ is compared to ${\bar{\text{C}}}_{\text{t}}^{\text{t}}$. After each iteration, v_max_m_ is adjusted in a direction until convergence of ${\bar{\text{C}}}_{\text{t}}$ to ${\bar{\text{C}}}_{\text{t}}^{\text{t}}$. To achieve this in practice, to reduce the computation time, the mean glucose concentration ${\bar{\text{C}}}_{\text{t}}$ is first computed over a grid of values v_max_m_. This function is then interpolated to get ${\bar{\text{C}}}_{\text{t}}$ as a function of v_max_m_. In the second step, the objective function $\left|{\bar{\text{C}}}_{\text{t}}\left({\text{v}}_{\text{max}\_\text{m}}\right)-{\bar{\text{C}}}_{\text{t}}^{\text{t}}\right|$ is minimized until its value is lower than 10^−9^. Having determined the specific value of v_max_m_, we use the previously interpolated C_t_(τ, v_max_m_) and *Q*(τ, C_t_(τ, v_max_m_)) to determine C_t_ (τ) and Q(τ) for any transit time τ. GEF, glucose extraction fraction; MTT, mean transit time; CTH, capillary transit time heterogeneity.

Figure 1 outlines our procedure for computing glucose extraction fraction (GEF) and CMR_glc_. Table 1 summarizes the different scientific questions asked in this study and the conditions under which the model is applied to answer each question. Note that the model is fully specified once the different input parameters in Table 1 are known. Table 2 summarizes the variables and quantities used in this computation.

**Table 1:** **Scientific questions raised in this study and corresponding conditions under which the model is applied**.

**Question**	**Parameter sets used**	**C_p_**	**${\bar{\text{C}}}_{\text{t}}$**	**MTT/CTH**	**Corresponding figure/location in the text**	**Supplementary information**
Q1: Does our model predict a linear relation between CBF and CMR_glc_ and between CBF and the tracer uptake, as it has been reported in the literature?	S_H_	Constant and equal to 500 μmol/100 mL.	bl: ${\bar{\text{C}}}_{\text{t}}$ is equal to 129 μmol/100 mL. ${\bar{\text{C}}}_{\text{t}}$ is linearly related to MTT such that it decreases by 30% between MTT_bl_ and MTT_st_	MTT and CTH in the range [MTT_bl_-MTT_st_] and [CTH_bl_-CTH_st_], respectively	Figure 2	
Q2: To what extent is CMR_glc_ affected when the plasma concentration vary from hypo- to hyperglycemic levels?	S_H_ and S_R_	In the range 0–3000 μmol/100 mL	${\bar{\text{C}}}_{\text{t}}$ is related to Cp according to Equations (13) and (14).	MTT = MTT_bl_; CTH = CTH_bl_	Figure 3	
Q3: To what extent MTT and CTH affect CMR_glc_? Comparison with CMRO_2_.	S_H_	Constant and equal to 500 μmol/100 mL.	bl: ${\bar{\text{C}}}_{\text{t}}$ is equal to 129 μmol/100 mL. ${\bar{\text{C}}}_{\text{t}}$ is linearly related to MTT such that it decreases by 30% between MTT_bl_ and MTT_st_.	MTT and CTH are in the range 0–2 s	Figure 4	CMR_glc_ is much less affected by changes in CTH than CMRO_2_. This can be seen from iso-contours which are much more vertical for CMR_glc_ than for CMRO_2_
Q4: Based on these predictions (Q3) for CMR_glc_ and CMRO_2_, what is the resulting OGI and lactate production?	S_H_	Constant and equal to 500 μmol/100 mL.	bl: ${\bar{\text{C}}}_{\text{t}}$ is equal to 129 μmol/100 mL (Equation 14). ${\bar{\text{C}}}_{\text{t}}$ is linearly related to MTT such that it decreases by 30% between MTT_bl_ and MTT_st_.	Take discrete values corresponding to the symbols (+) and (×) on Figure 4.	Figure 5	Note that ${\bar{\text{C}}}_{\text{t}}$ during baseline is calibrated to yield an OGI equal to 5.5 at baseline
Q5: Applying our model to glucose tracer (FDG), how is the LC predicted to vary as a function of the plasma concentration and between physiological states ? Are these predictions in agreement with the literature?	S_H_ and S_R_	In the range 0–3000 μmol/100 mL	bl: ${\bar{\text{C}}}_{\text{t}}$ is related to Cp according to Equations (13) and (14). st: ${\bar{\text{C}}}_{\text{t}}$ is assumed to be decreased by 30% compared its value at baseline	bl: MTT = MTT_bl_; CTH = CTH_bl_ st: MTT = MTT_st_; CTH = CTH_st_	Figure 6	
Q6: How much would the overestimation in CMR_glc_ be when neglecting the changes in LC?	S_H_	Constant and equal to 500 μmol/100 mL.	bl: ${\bar{\text{C}}}_{\text{t}}$ is set to 132 μmol/100 mL (Equation 14) and decreases by a value between 0 and 40% during stimulation	bl: MTT = MTT_bl_; CTH = CTH_bl_ st: MTT = MTT_st_; CTH = CTH_st_	Figure 7	The overestimation in CMR_glc_ increase is calculated using (Equation 28), which relates the relative changes in CMR_glc_, in CMR_glc_, app, and LC
Q7: What prediction can we make for the metabolism and the lumped constant when we apply the model to tumor cells?	S_H_	Constant and equal to 500 μmol/100 mL	${\bar{\text{C}}}_{\text{t}}$ during baseline is set to 132 μmol/100 mL initially and decreases as v_max_m_ increases.	MTT = MTT_bl_; CTH = CTH_bl_	Discussion (see Section Applying Our Model to Disease Conditions)	

*S_H_: parameter set when the model is applied to humans: v_max_t_ = 68 μmol/100 mL_brain/min and K_T_ = 50 μmol/100 mL_brain. S_R_: parameter set when the model is applied to rats: v_max_t_ = 136 μmol/100 mL_brain/min and K_T_ = 150 μmol/100 mL_brain. bl, baseline; st, stimulation. MTT_bl_ = 1.4 s; MTT_st_ = 0.81 s; CTH_bl_ = 1.33 s; CTH_st_ = 0.52 s*.

**Table 2:** **Terminology and parameter values for glucose extraction model**.

**Symbol**	**Name, definition**	Scale^*^: single capillary level/network level	**Value**	**Unit**
C_A_	Arterial glucose concentration in plasma	Network		μmol/100 mL_plasma
C_A_′	Arterial glucose tracer concentration in plasma	Network	<0.5	μmol/100 mL_plasma
CMR_glc_	Glucose metabolism	Network		μmol/100 mL_brain/min
Cp	Glucose concentration in plasma	Single capillary	Varies along the capillary axis	μmol/100 mL_plasma
Cp′	Glucose tracer concentration in plasma	Single capillary	Varies along the capillary axis	μmol/100 mL_plasma
Ct	Glucose concentration in extravascular tissue	Single capillary		μmol/100 mL_brain
${\bar{\text{C}}}_{\text{t}}^{\text{t}}$	Glucose concentration in extravascular tissue. Target value (input)	Network		μmol/100 mL_brain
${\bar{\text{C}}}_{\text{t}}$	Glucose concentration in extravascular tissue	Network	${\bar{\text{C}}}_{\text{t}}$ = ${\bar{\text{C}}}_{\text{t}}^{\text{t}}$	μmol/100 mL_brain
CTH	Capillary transit time heterogeneity	Network		Second
GEF	Glucose extraction fraction	Network		
h	Capillary transit time distribution	Network		1/s
$\tilde{\text{h}}$	Capillary transit time distribution	Network		1/s
K_M_	Michaelis-Menten parameter for glucose metabolism	Network	5	μmol/100 mL_brain
K_M_'	Michaelis-Menten parameter for glucose tracer phosphorylation	Network	5	μmol/100 mL_brain
K_T_	Michaelis-Menten parameter for glucose transport across the capillary membrane	Network	Parameter set S_H_: 50 Parameter set S_R_:150	μmol/100 mL_brain
K_T_'	Michaelis-Menten parameter for glucose tracer transport across the capillary membrane	Network	K_T_' = K_T_	μmol/100 mL_brain
LC	Lumped constant (GEF/GEF' in steady state)	Network		No unit
M	Glucose metabolism	Single capillary		μmol/100 mL_brain/min
M'	Glucose tracer metabolism phosphorylation rate	Single capillary		μmol/100 mL_brain/min
MTT	Mean transit time	Network		Second
Q	Glucose extraction fraction	Single capillary		No unit
Q'	Glucose tracer extraction fraction	Single capillary		No unit
V_cap_	Cerebral capillary volume	Network	1.4	mL/100 mL_brain
V_d_	Physical distribution space of glucose in the brain	Network	0.77	mL_accessible_extra-vascular_tissue/mL_brain
V_d_'	Physical distribution space of glucose tracer in the brain	Network	0.77	mL_accessible_extra-vascular_tissue/mL_brain
v_max_m_	Effective maximum rate at which glucose is phosphorylated by hexokinase	Network	30 in the resting state with parameter set S_H_	μmol/100 mL_brain/min
v_max_m_'	Effective maximum rate at which glucose tracer is phosphorylated by hexokinase	Network	0.3·v_max_m_	μmol/100 mL_brain/min
v_max_t_	Maximum rate at which glucose is transported across the capillary membrane	Network	Parameter set S_H_: 68 Parameter set S_R_: 136	μmol/100 mL_brain/min
v_max_t_'	Maximum rate at which glucose tracer is transported across the capillary membrane	Network	1.4·v_max_t_	μmol/100 mL_brain/min
τ	Transit time	Single capillary		Second

* *Network level refers to parameters with same value for each capillary of the network, or to quantities than can only be defined at this level. Single capillary level refers to parameters which can take different values between capillaries*.

Our model aims to determine CMR_glc_ in steady state from MTT, CTH, and the mean glucose concentration in the tissue ${\bar{\text{C}}}_{\text{t}}^{\text{t}}$, where the superscript t stands for “target value.” CMR_glc_ is derived directly from GEF using the formula CMR_glc_ = C_A_ · CBF · GEF (Fick's principle) where C_A_ is the arterial glucose concentration, and from the central volume theorem which relates CBF to the MTT and the capillary volume V_cap_ through the relation CBF = V_cap_/MTT.

To compute the mean value of any function over the capillary network, we sum the contribution of the function for every capillary, weighted by the transit time distribution h(τ). In particular, GEF corresponds simply to the mean of the single capillary glucose extraction fraction Q:

(1) $$\text{GEF}\left(\text{MTT},\text{CTH}\right)=\bar{\text{Q}}=\int_{0}^{+\infty }\text{d}\tau \;\cdot \text{ Q}\left(\tau \right)\;\cdot \text{ h}\left(\tau ,\text{MTT},\text{CTH}\right)$$

The transit time distribution *h*(τ) is assumed to be a gamma distribution. This choice has been discussed extensively in Angleys et al. (2015). The gamma distribution is a two parameter distribution and is therefore fully and uniquely specified through the dependence on its mean (MTT) and standard deviation (CTH).

The individual steps to determine Q as a function of τ in order to determine GEF with Equation (1) are explained in details below. To simplify the notation, we will not indicate the dependence of the different parameters on MTT and CTH, which are fixed during the entire procedure.

### Derivation of the system equations

#### Network level

At the capillary network level, the mean glucose concentration in the tissue is given and equal to ${\bar{\text{C}}}_{\text{t}}^{\text{t}}$. In this model, we assume that glucose transfer among capillaries is negligible. As a result, at the capillary level, glucose concentrations C_t_ are not necessarily identical around the capillaries in our tissue compartment, and glucose concentration in tissue may therefore vary at the intercapillary distance scale.

Tissue concentrations in each compartment C_t_ must fulfill the equation:

(2) $${\bar{\text{C}}}_{\text{t}}^{\text{t}}-\int_{0}^{+\infty }\text{d}\tau \;\cdot \;\tilde{\text{h}}\left(\tau \right)\;\cdot \;{\text{C}}_{\text{t}}\left(\tau ,{\text{v}}_{\max\_\text{m}}\right)=0$$

where v_max_m_ is a constant which will be determined later. $\tilde{\text{h}}$ is derived from h, according to the relation:

(3) $$\tilde{\text{h}}\left(\tau \right)=\text{h}\left(\tau \right)\;\cdot \;\frac{\tau }{\text{MTT}}$$

Here, $\tilde{\text{h}}\left(\tau \right)d\tau$ represents a volume fraction of capillaries, as opposed to a fraction of the flow (implicit for h). Please see the Supplementary Material for more details concerning the derivation of this distribution.

#### Capillary scale

##### Equilibrium concentration in the tissue compartment

At the capillary scale, in steady state, there is no glucose accumulation in the tissue compartment and the net rate of glucose uptake from the plasma equals the rate M at which glucose is phosphorylated by hexokinase:

(4) $$\frac{{\text{C}}_{\text{A}}\;\cdot \text{ Q}\left(\tau ,{\text{C}}_{\text{t}}\right)}{\tau }\;\cdot \;{\text{V}}_{\text{cap}}-\text{M}\left(\tau ,{\text{C}}_{\text{t}},{\text{v}}_{\max\_\text{m}}\right)=0$$

where C_A_ · Q(τ, C_t_) · V_cap_/τ represents the net flux of glucose across a single capillary membrane, with C_A_ being the arterial glucose concentration in plasma, Q the extraction fraction for a single capillary and the capillary transit time. V_cap_ is assumed to be constant and equal to 1.4%.

We assume that M is governed by Michaelis-Menten kinetics (Michaelis and Menten, 1913):

(5) $$\text{M }\left(\tau ,{\text{C}}_{\text{t}},{\text{v}}_{\max\_\text{m}}\right)={\text{v}}_{\max\_\text{m}}\;\cdot \;\frac{\frac{{\text{C}}_{\text{t}}\left(\tau ,{\text{v}}_{\max\_\text{m}}\right)}{{\text{V}}_{\text{d}}}}{{\text{K}}_{\text{M}}+\frac{{\text{C}}_{\text{t}}\left(\tau ,{\text{v}}_{\max\_\text{m}}\right)}{{\text{V}}_{\text{d}}}}$$

where K_M_ is a parameter such that V_d_ · K_M_ is the concentration at which metabolism equals v_max_m_/2. v_max_m_ is the effective maximum rate at which hexokinase can metabolize glucose to glucose-6-phosphate.

Hence, Equation (4) can be rewritten:

(6) $$\frac{{\text{C}}_{\text{A}}\;\cdot \text{ Q}\left(\tau ,{\text{C}}_{\text{t}}\right)}{\tau }\;\cdot \;{\text{V}}_{\text{cap}}-{\text{v}}_{\max\_\text{m}}\;\cdot \;\frac{\frac{{\text{C}}_{\text{t}}\left(\tau ,{\text{v}}_{\max\_\text{m}}\right)}{{\text{V}}_{\text{d}}}}{{\text{K}}_{\text{M}}+\frac{{\text{C}}_{\text{t}}\left(\tau ,{\text{v}}_{\max\_\text{m}}\right)}{{\text{V}}_{\text{d}}}}=0$$

##### Glucose extraction fraction from the plasma

In this section, we detail how we derive the equations to express the glucose extraction fraction for a single capillary as a function of the transit time and of the concentration gradient. Glucose is considered in two compartments: plasma and extravascular tissue. Glucose is transported across the capillary membrane by facilitated diffusion via the glucose transporter GLUT-1, and the unidirectional flux of glucose across the BBB is assumed to be governed by reversible Michaelis-Menten kinetics (Cunningham, 1986). We assume that within the capillary, axial diffusion can be neglected compared to advective transport. Considering only steady-state conditions and choosing the z-axis to be oriented along the capillary, we let C_p_(z) and C_t_ denote plasma and tissue glucose concentration, respectively. The system is then described by the following differential equation:

(7) $$\frac{\partial {\text{C}}_{\text{p}}\left(\text{x},\tau ,{\text{C}}_{\text{t}}\right)}{\partial \text{x}}=-\frac{\tau }{{\text{V}}_{\text{cap}}}\;\cdot \;{\text{v}}_{\max\_\text{t}}\;.\;\frac{{\text{C}}_{\text{p}}\left(\text{x},\tau ,{\text{C}}_{\text{t}}\right)-\frac{{\text{C}}_{\text{t}}\left(\tau ,{\text{v}}_{\max\_\text{m}}\right)}{{\text{V}}_{\text{d}}}}{{\text{K}}_{\text{T}}+{\text{C}}_{\text{p}}\left(\text{x},\tau ,{\text{C}}_{\text{t}}\right)+\frac{{\text{C}}_{\text{t}}\left(\tau ,{\text{v}}_{\max\_\text{m}}\right)}{{\text{V}}_{\text{d}}}}$$

Here,

$$-\frac{\tau }{{\text{V}}_{\text{cap}}}\;\cdot \;{\text{v}}_{\max\_\text{t}}\;.\;\frac{{\text{C}}_{\text{p}}\left(\text{x},\tau ,{\text{C}}_{\text{t}}\right)}{{\text{K}}_{\text{T}}+{\text{C}}_{\text{p}}\left(\text{x},\tau ,{\text{C}}_{\text{t}}\right)+\frac{{\text{C}}_{\text{t}}\left(\tau ,{\text{v}}_{\max\_\text{m}}\right)}{{\text{V}}_{\text{d}}}}$$

corresponds to glucose efflux from the plasma to the tissue, while:

$$\frac{\tau }{{\text{V}}_{\text{cap}}}\;\cdot \;{\text{v}}_{\max\_\text{t}}\;.\;\frac{\frac{{\text{C}}_{\text{t}}\left(\tau ,{\text{v}}_{\max\_\text{m}}\right)}{{\text{V}}_{\text{d}}}}{{\text{K}}_{\text{T}}+{\text{C}}_{\text{p}}\left(\text{x},\tau ,{\text{C}}_{\text{t}}\right)+\frac{{\text{C}}_{\text{t}}\left(\tau ,{\text{v}}_{\max\_\text{m}}\right)}{{\text{V}}_{\text{d}}}}$$

corresponds to glucose influx, back from the tissue to the plasma.

In equation (7), x ∈ [0;1] is a normalized axial coordinate, i.e., x = z/L, with L being the capillary length; V_d_ is the physical distribution space of glucose in the brain, equal to 0.77 (Lund-Andersen, 1979). Throughout this study, the two Michaelis-Menten parameters, namely v_max_t_, which corresponds to the maximum rate at which glucose can be transported unidirectionally across the BBB, and K_T_, have been assigned values as explained in the Section Calibration of the Model Parameters. The plasma glucose concentration at the point x = 0 is assumed to be equal to glucose arterial plasma concentration C_A_. The glucose extraction fraction for a single capillary is defined by the ratio

(8) $$\text{Q}\left(\tau ,{\text{C}}_{\text{t}}\right)=\frac{{\text{C}}_{\text{p}}\left(0,\tau ,{\text{C}}_{\text{t}}\right)-{\text{C}}_{\text{p}}\left(1,\tau ,{\text{C}}_{\text{t}}\right)}{{\text{C}}_{\text{p}}\left(0,\tau ,{\text{C}}_{\text{t}}\right)}$$

and depends on the transit time τ.

Summarizing, we must solve the following system of coupled equations:

(9) $$\left\{ \begin{array}{l} (\text{a})\;{\bar{\text{C}}}_{\text{t}}^{\text{t}}-\int_{0}^{+\infty }\tilde{\text{h}}\left(\tau \right)\;\cdot \;{\text{C}}_{\text{t}}\left(\tau ,{\text{v}}_{\max\_\text{m}}\right)\;\cdot \;d\tau =0\\ (\text{b})\frac{-{\text{v}}_{\max\_\text{m}}\;\cdot \;\frac{{\text{C}}_{\text{t}}\left(\tau ,{\text{v}}_{\max\_\text{m}}\right)}{{\text{V}}_{\text{d}}}}{{\text{K}}_{\text{M}}+\frac{{\text{C}}_{\text{t}}\left(\tau ,{\text{v}}_{\max\_\text{m}}\right)}{{\text{V}}_{\text{d}}}}+\frac{{\text{C}}_{\text{A}}\;\cdot \;Q\left(\tau ,{\text{C}}_{\text{t}}\left(\tau ,{\text{v}}_{\max\_\text{m}}\right)\right)}{\tau }\cdot \\ \;{\text{V}}_{\text{cap}}=0\text{,}\\ \text{with Q}\left(\tau ,{\text{C}}_{\text{t}}\left(\tau ,{\text{v}}_{\max\_\text{m}}\right)\right)=1-\frac{{\text{C}}_{\text{p}}\left(1,\tau ,{\text{C}}_{\text{t}}\left(\tau ,{\text{v}}_{\max\_\text{m}}\right)\right)}{{\text{C}}_{\text{p}}\left(0,\tau ,{\text{C}}_{\text{t}}\left(\tau ,{\text{v}}_{\max\_\text{m}}\right)\right)}\\ (\text{c})\frac{\partial {\text{C}}_{\text{p}}\left(\text{x},\tau ,{\text{C}}_{\text{t}}\left(\tau ,{\text{v}}_{\max\_\text{m}}\right)\right)}{\partial x}=-\frac{\tau }{{\text{V}}_{\text{cap}}}\cdot \\ \;{\text{v}}_{\max\_\text{t}}\;.\;\frac{{\text{C}}_{\text{p}}\left(\text{x},\tau ,{\text{C}}_{\text{t}}\left(\tau ,{\text{v}}_{\max\_\text{m}}\right)\right)-\frac{{\text{C}}_{\text{t}}\left(\tau ,{\text{v}}_{\max\_\text{m}}\right)}{{\text{V}}_{\text{d}}}}{{\text{K}}_{\text{T}}+{\text{C}}_{\text{p}}\left(\text{x},\tau ,{\text{C}}_{\text{t}}\left(\tau ,{\text{v}}_{\max\_\text{m}}\right)\right)+\frac{{\text{C}}_{\text{t}}\left(\tau ,{\text{v}}_{\max\_\text{m}}\right)}{{\text{V}}_{\text{d}}}}\text{,}\\ \text{with }{\text{C}}_{\text{p}}\left(0,\tau ,{\text{C}}_{\text{t}}\left(\tau ,{\text{v}}_{\max\_\text{m}}\right)\right)={\text{C}}_{\text{A}} \end{array}\right.$$

for C_p_, C_t_ and v_max_m_, for any value of the transit time τ and relevant values of ${\bar{\text{C}}}_{\text{t}}^{\text{t}}$. The computation is performed numerically in several steps, as no analytical solution exists for this system.

### Solving the equation system

In this section, we detail the steps to solve system (9). Briefly, in the first step, we will use Equation (9)c to express Q as a function of τ and C_t_. In the second step, we will use Equation (9)b to express C_t_ as a function of τ and v_max_m_. Finally, we use Equation (9)a to determine the value of v_max_m_ to get explicitly Q as a function of τ.

In the first step, we solve Equation (9)c independently over a grid of values (τ, C_t_). We compute the corresponding Q function on the same grid:

(10) $$\text{Q}\left(\tau ,{\text{C}}_{\text{t}}\right)=1-\frac{{\text{C}}_{\text{p}}\left(1,\tau ,{\text{C}}_{\text{t}}\right)}{{\text{C}}_{\text{p}}\left(0,\tau ,{\text{C}}_{\text{t}}\right)}$$

This function is then appropriately interpolated to get sufficiently high resolution of Q(τ, C_t_) while minimizing the amount of numerical computation.

In the second step, we numerically solve the equation:

(11) $$-{\text{v}}_{\max\_\text{m}}\;\cdot \;\frac{\frac{{\text{C}}_{\text{t}}\left(\tau ,{\text{v}}_{\max\_\text{m}}\right)}{{\text{V}}_{\text{d}}}}{{\text{K}}_{\text{M}}+\frac{{\text{C}}_{\text{t}}\left(\tau ,{\text{v}}_{\max\_\text{m}}\right)}{{\text{V}}_{\text{d}}}}+\frac{{\text{C}}_{\text{A}}\;\cdot \text{ Q}\left(\tau ,{\text{C}}_{\text{t}}\left(\tau ,{\text{v}}_{\max\_\text{m}}\right)\right)}{\tau }\;\cdot \;{\text{V}}_{\text{cap}}=0$$

for C_t_(τ, v_max_m_) over an array of values v_max_m_, and for relevant values of τ, to obtain C_t_ (τ, v_max_m_) in steady-state on the same grid (τ, v_max_m_). This function is then appropriately interpolated.

The last step consists in solving numerically the equation:

(12) $${\bar{\text{C}}}_{\text{t}}^{\text{t}}-\int_{0}^{+\infty }\text{d}\tau \;\cdot \;\tilde{\text{h}}\left(\tau \right)\;\cdot \;{\text{C}}_{\text{t}}\left(\tau ,{\text{v}}_{\max\_\text{m}}\right)=0$$

in order to determine the v_max_m_ which fulfills this relation.

Having determined v_max_m_, we use the previously interpolated C_t_ (τ, v_max_m_) and Q (τ, C_t_ (τ, v_max_m_)) to determine C_t_ (τ) and Q(τ) for any transit time.

### Predicted changes in OGI during functional activation based on experimental data from the literature

We combine CMR_glc_ values with CMRO_2_ values obtained with our previous model of oxygen extraction (Angleys et al., 2015) to predict the extent to which the OGI changes as a function of MTT, CTH and physiological state. Transit time characteristics in baseline and during activation are obtained from *in vivo* rat studies by Schulte et al. (2003) and Stefanovic et al. (2008). They appear as symbols on CMR_glc_ and CMRO_2_ maps of **Figure 4** and are listed in Table 2 in Angleys et al. (2015). We predict and compare CMRO_2_ and CMR_glc_ in the different physiological states, and evaluate their dependence on the transit time distribution. The model of oxygen extraction is used under the assumption that the maximum metabolic rate of oxygen v_max_ increases by 10% from baseline condition to stimulation. See discussion in Angleys et al. (2015). Note, however, that in this latter model, CMRO_2_ itself is predicted to increase by about 20% between baseline and stimulation.

### Calibration of the model parameters

The model parameters K_M_, v_max_t_, K_T_, v_max_m_ as well as the input function ${\bar{\text{C}}}_{\text{t}}^{\text{t}}$ must be fixed based on literature values and realistic assumptions. K_M_ was inferred from literature reports, and we set its value to 5 μmol/100 mL_brain (McIlwain and Bachelard, 1985). We tested our model using two different sets of Michaelis-Menten parameters to describe glucose transport. One parameter set, S_R_, was obtained by calibrating our model to two studies conducted in rats. The first (Cunningham et al., 1986) utilized an intravenous infusion technique in rats and the second study (Choi et al., 2001) involves NMRS in rats. While CMR_glc_ is reported in the first study, we assume that it is equal to 45μmol/100mL/min (Choi and Gruetter, 2012) in the second, where α-chloralose-anesthetized rats were used. We set v_max_t_ to 136μmol/100mL/min, the mean of the value reported in the two studies.

The second parameter set, S_H_, was obtained by calibrating our model to two studies involving NMRS in human (Gruetter et al., 1998; Seaquist et al., 2001). We assumed CMR_glc_ = 30μmol/100*mL*/min in these two studies and set v_max_t_ to 68μmol/100mL/min.

The values reported for K_T_ vary considerably between studies, even when identical protocols are used (see for example, Choi et al., 2001; Seaquist et al., 2001, for reported values). Even negative values have been reported (Seaquist et al., 2001), although the physical meaning of such findings remain unclear. As a result, the calibration of K_T_ is somewhat uncertain. When predicting CMR_glc_ as a function of plasma concentration (**Figure 3**), K_T_ primarily influences the plasma concentration at which the metabolism reaches its asymptotical value, that is, the slope of the curve in **Figure 3**. Several reports suggest that CMR_glc_ is relatively independent of plasma concentration for concentrations ranging between mild hypoglycemic and hyperglycemic levels (Bryan et al., 1986; Orzi et al., 1988; Suda et al., 1990; Hasselbalch et al., 2001b). Accordingly, we chose K_T_ equal to 50 μ*mol/100 mL* and 150 μ*mol/100 mL* when the model was applied to humans and rats, respectively. These values are of the same order of magnitude as those often reported in the literature.

Several reports suggest that the phosphorylation catalyzed by hexokinase is an important control step in the regulation of glucose metabolism in the brain (Clarke et al., 1989). In resting conditions, hexokinase is strongly inhibited, in particular by its product, glucose-6-phosphate, so that its operation rate at rest is only 3–10% of its maximum value (Clarke et al., 1989), suggesting that glucose metabolism can increase to accommodate higher energy demand. Accordingly, v_max_m_ is calibrated in this model to yield the glucose tissue concentration target value ${\bar{\text{C}}}_{\text{t}}^{\text{t}}$, as described in the previous section (Solving the Equation System). Consequently, v_max_m_ is the maximum rate at which hexokinase can metabolize glucose to glucose-6-phosphate when inhibited, and is therefore the *effective* maximum rate of hexokinase.

In the following, the state (MTT_bl_ = 1.4 s, CTH_bl_ = 1.33 s) is taken as a reference for resting state, while the state (MTT_st_ = 0.81 s, CTH_st_ = 0.52 s) is taken as a reference for stimulation, in accordance with an experiment involving functional activation in rat (Stefanovic et al., 2008), and two studies modeling cerebral oxygen consumption using these same states as a reference for resting state and stimulation (Jespersen and Østergaard, 2012; Angleys et al., 2015). We will refer to these two states as resting state or baseline condition, and stimulation or activation, respectively.

#### ${\bar{\text{C}}}_{\text{t}}^{\text{t}}$ in baseline condition

Part of this study aims to determine the extent to which CMR_glc_ and LC are predicted to change as a function of the plasma concentration (see **Figures 3**, **6**). For this part of the study, we assumed that ${\bar{\text{C}}}_{\text{t}}^{\text{t}}$ in the baseline condition varies with plasma concentration according to measurements reported in two studies, where plasma concentrations ranged from hypoglycemic to hyperglycemic levels. The first study reported direct measurements of glucose concentration in cerebral tissue in conscious rats (Dienel et al., 1991) and the second study was based on NMRS measurements in humans (Gruetter et al., 1998). Arterial and tissue concentrations were thus assumed to obey relations:

(13) $${\bar{\text{C}}}_{\text{t}}^{\text{t}}=\{\begin{array}{c} 0.24\;\cdot \;{\text{C}}_{\text{A}}-72\text{ if }{\text{C}}_{\text{A}}>400\;\mu \text{mol}/100\text{ mL}\_\text{ plasma}\\ 24\;\cdot \;\frac{\text{exp }\left({\text{C}}_{\text{A}}/102\;\right)-1}{\text{exp }\left(400/102\;\right)-1}\text{ otherwise} \end{array}$$

(14) $${\bar{\text{C}}}_{\text{t}}^{\text{t}}=\{\begin{array}{c} 0.30\;\cdot \;{\text{C}}_{\text{A}}-18\text{ if }{\text{C}}_{\text{A}}>250\;\mu \text{mol}/100\text{ mL}\_\text{ plasma}\\ \;57\;\cdot \;\frac{\text{exp }\left({\text{C}}_{\text{A}}/434\;\right)-1}{\text{exp }\left(250/434\;\right)-1}\text{ otherwise} \end{array}$$

when the model was applied to rats and humans, respectively.

In Equations (13) and (14), ${\bar{\text{C}}}_{\text{t}}^{\text{t}}$ is expressed in μmol/100 mL_brain and C_A_ in μmol/100 mL_plasma. Another part of the study aims to quantify the extent to which CMR_glc_ and the OGI change between physiological conditions, under condition of fixed plasma concentration (**Figures 4**, **5**) and when the model is applied to human. We fixed ${\bar{\text{C}}}_{\text{t}}^{\text{t}}$ and C_A_ such that the molar ratio CMRO_2_:CMR_glc_ in the resting state (MTT = 1.4 s, CTH = 1.33 s) is equal to 5.5, as reported in Madsen et al. (1995) and Hasselbalch et al. (1996b). In this state, we assume CMRO_2_ to be 3.8μmol/100 mL_brain/min (i.e., 158 μmol/100 mL_brain/min), as in Jespersen and Østergaard (2012) and Angleys et al. (2015). Accordingly, we set ${\bar{\text{C}}}_{\text{t}}^{\text{t}}$ to 129 μmol/100 mL_brain and C_A_ = 500μmol/100 mL_plasma, which yields a corresponding CMR_glc_ in baseline condition equal to 29 μmol/100 mL/min.

#### ${\bar{\text{C}}}_{\text{t}}^{\text{t}}$ during stimulation

Based on experimental data, we assume that ${\bar{\text{C}}}_{\text{t}}^{\text{t}}$ decreases by 30% from baseline condition to stimulation. For states intermediate between baseline and stimulation, we assume that ${\bar{\text{C}}}_{\text{t}}^{\text{t}}$ is a function of MTT only, and we determine its value by linear interpolation. We also assume that the linear relation between MTT and ${\bar{\text{C}}}_{\text{t}}^{\text{t}}$ is preserved for MTT outside of the interval [MTT_bl_ MTT_st_]. These assumptions are discussed further below.

### Glucose tracer kinetics and the lumped constant

We use our model to predict the value of the LC, which corresponds to the ratio between glucose analog (tracer) and native glucose extraction in steady state. In the following, models were calibrated to predict the LC for FDG, and “glucose analog” therefore refers to FDG throughout the manuscript unless otherwise specified. Accordingly, we determine the mean extraction fraction of glucose tracer (GEF′) over the capillary network. GEF′ is determined as GEF previously:

(15) $${\text{GEF}}^{\prime }=\int_{0}^{+\infty }\text{d}\tau \;\cdot \;{\text{Q}}^{\prime }\left(\tau \right)\;\cdot \text{ h}\left(\tau \right)$$

where Q′ is the single capillary glucose analog extraction fraction that we must determine.

In steady state, there is no accumulation of unphosphorylated glucose analog in the extravascular compartment and the net rate of glucose analog uptake from the plasma equals the rate at which glucose is phosphorylated.

Hence, at the single capillary level:

(16) $$\frac{{\text{C}}_{\text{A}}\prime \;\cdot \;{\text{Q}}^{\prime }\left(\tau ,{\text{C}}_{\text{t}},{\text{C}}_{\text{t}}\prime \right)}{\tau }\;\cdot \;{\text{V}}_{\text{cap}}-\text{M}\prime \left({\text{C}}_{\text{t}},{\text{C}}_{\text{t}}\prime ,{\text{v}}_{\max\_\text{m}},{\text{v}}_{\max\_\text{m}}\prime \right)=0$$

Where M′ is the rate at which glucose tracer is phosphorylated and ${\text{C}}_{\text{A}}^{\prime }$ is the arterial glucose analog concentration.

#### Single capillary scale

The glucose analog is assumed to follow the same kinetics as native glucose, that is:

(17) $$\begin{array}{l} {\text{M}}^{\prime }\left({\text{C}}_{\text{t}},{\text{C}}_{\text{t}}\prime ,{\text{v}}_{\max\_\text{m}},{\text{v}}_{\max\_\text{m}}\prime \right)\\ \;={\text{v}}_{\max\_\text{m}}\prime \cdot \frac{{\text{C}}_{\text{t}}\prime \left(\tau ,{\text{v}}_{\max\_\text{m}},{\text{v}}_{\max\_\text{m}}\prime \right)/{\text{V}}_{\text{d}}\prime \;}{{\text{K}}_{\text{M}}\prime \;\cdot \;\left(1+\frac{{\text{C}}_{\text{t}}/{\text{V}}_{\text{d}}\;}{{\text{K}}_{\text{M}}}\right)+\frac{{\text{C}}_{\text{t}}\prime }{{\text{V}}_{\text{d}}\prime }} \end{array}$$

With v_max_m_′ being the effective maximum rate at which glucose analog can be phosphorylated, C_t_′ the concentration of glucose analog in the tissue, V_d_′ the physical distribution space of glucose analog in the brain, and ${{\text{K}}_{\text{M}}}^{\prime }$ such that ${\text{V}}_{\text{d}}\cdot {{\text{K}}_{\text{M}}}^{\prime }$ is the concentration at which ${\text{M}}^{\prime }={{\text{v}}_{\max\text{\_}\text{m}}}^{\prime }/2$ under condition no native glucose in the tissue (C_t_ = 0). In the following, we set ${{\text{v}}_{\max\text{\_}\text{m}}}^{\prime }=0.3\cdot {\text{v}}_{\max\text{\_}\text{m}}$ (Kuwabara et al., 1990; Kuwabara and Gjedde, 1991). Equation (17) can be simplified to:

$${\text{M}}^{\prime }\left({\text{C}}_{\text{t}},{\text{v}}_{\max\_\text{m}}\right)={\text{v}}_{\max\_\text{m}}\prime \;\cdot \;\frac{{\text{C}}_{\text{t}}\prime \left(\tau ,{\text{v}}_{\max\_\text{m}}\right)/{\text{V}}_{\text{d}}\prime \;}{{\text{K}}_{\text{M}}+{\text{C}}_{\text{t}}\left(\tau ,{\text{v}}_{\max\_\text{m}}\right)/{\text{V}}_{\text{d}}\;}$$

in the limit where C_t_ is much greater than ${\text{C}}_{\text{t}}^{\prime }$ and setting ${{\text{K}}_{\text{M}}}^{\prime }$ = K_M_, assuming identical hexokinase affinities for the two substrates. Note that, because of the concentrations involved, glucose analog transport is influenced by native glucose concentration, whereas the contrary is not true.

##### Extraction fraction from the plasma

We consider glucose analog in the same two compartments as native glucose, and we make the same assumptions to determine its concentration along the capillary axis:

(18) $$\begin{array}{l} \frac{\partial {\text{C}}_{\text{p}}\prime \left(\text{x},\tau ,{\text{C}}_{\text{t}},{\text{C}}_{\text{t}}\prime \right)}{\partial \text{x}}=-\frac{\tau }{{\text{V}}_{\text{cap}}}\;\cdot \;{\text{v}}_{\max\_\text{t}}\prime \cdot \\ \frac{{\text{C}}_{\text{p}}\prime \left(\text{x},\tau ,{\text{C}}_{\text{t}},{\text{C}}_{\text{t}}\prime \right)-\frac{{\text{C}}_{\text{t}}\prime \left(\tau ,{\text{v}}_{\max\_\text{m}}\right)}{{\text{V}}_{\text{d}}\prime }}{{\text{K}}_{\text{T}}\prime \;\cdot \;\left(1+\frac{{\text{C}}_{\text{p}}\left(\text{x},\tau ,{\text{C}}_{\text{t}}\right)}{{\text{K}}_{\text{T}}}+\frac{{\text{C}}_{\text{t}}\left(\tau ,{\text{v}}_{\max\_\text{m}}\right)}{{\text{V}}_{\text{d}}\;\cdot \;{\text{K}}_{\text{T}}}\right)+{\text{C}}_{\text{p}}\prime \left(\text{x},\tau ,{\text{C}}_{\text{t}},{\text{C}}_{\text{t}}\prime \right)+\frac{{\text{C}}_{\text{t}}\prime \left(\tau ,{\text{v}}_{\max\_\text{m}}\right)}{{\text{V}}_{\text{d}}\prime }} \end{array}$$

Where ${\text{C}}_{\text{p}}^{\prime }$ denotes the glucose analog concentration in plasma, ${{\text{v}}_{\max\text{\_}\text{t}}}^{\prime }$ the maximum rate at which it can be transported across the capillary membrane, and ${\text{K}}_{\text{T}}^{\prime }$ the Michaelis-Menten parameters for glucose tracer transport. The glucose analog concentration at the point x = 0 is equal to glucose tracer arterial concentration ${\text{C}}_{\text{A}}^{\prime }$, which is assumed to be negligible compared to C_A_ (tracer concentration).

Hence, Equation (18) can be simplified

(19) $$\begin{array}{l} \frac{\partial {\text{C}}_{\text{p}}\prime \left(\text{x},\tau ,{\text{C}}_{\text{t}},{\text{C}}_{\text{t}}\prime \right)}{\partial \text{x}}=\\ -\frac{\tau }{{\text{V}}_{\text{cap}}}\;\cdot \;{\text{v}}_{\max\_\text{t}}\prime \;.\;\frac{{\text{C}}_{\text{p}}\prime \left(\text{x},\tau ,{\text{C}}_{\text{t}},{\text{C}}_{\text{t}}\prime \right)-\frac{{\text{C}}_{\text{t}}\prime \left(\tau ,{\text{v}}_{\max\_\text{m}}\right)}{{\text{V}}_{\text{d}}\prime }}{{\text{K}}_{\text{T}}\prime \;\cdot \;\left(1+\frac{{\text{C}}_{\text{p}}\left(\tau ,{\text{C}}_{\text{t}}\right)}{{\text{K}}_{\text{T}}}+\frac{{\text{C}}_{\text{t}}\left(\tau ,{\text{v}}_{\max\_\text{m}}\right)}{{\text{V}}_{\text{d}}\;\cdot \;{\text{K}}_{\text{T}}}\right)} \end{array}$$

Where we set ${{\text{v}}_{\max\text{\_}\text{t}}}^{\prime }=1.4\cdot {\text{v}}_{\max\text{\_}\text{t}}$ (Crane et al., 1983; Kuwabara et al., 1990; Hasselbalch et al., 1996a), and approximate in the following ${{\text{K}}_{\text{T}}}^{\prime }$ and ${{\text{V}}_{\text{d}}}^{\prime }$ to be equal to K_T_ and V_d_, respectively.

Hence, for glucose analog, we must solve the following system of coupled equation:

(20) $$\left\{ \begin{array}{l} (\text{a})-{\text{v}}_{\max\_\text{m}}\prime \;\cdot \;\frac{{\text{C}}_{\text{t}}\prime \left(\tau ,{\text{v}}_{\max\_\text{m}}\right)/{\text{V}}_{\text{d}}\;}{{\text{K}}_{\text{M}}+\frac{{\text{C}}_{\text{t}}\left(\tau ,{\text{v}}_{\max\_\text{m}}\right)}{{\text{V}}_{\text{d}}}}+\frac{{\text{C}}_{\text{A}}\prime \;\cdot \;{\text{Q}}^{\prime }\left(\tau ,{\text{C}}_{\text{t}},{\text{C}}_{\text{t}}\prime \right)}{\tau }\cdot \\ \;{\text{V}}_{\text{cap}}=0,\text{ with }{\text{Q}}^{\prime }\left(\tau ,{\text{C}}_{\text{t}},{\text{C}}_{\text{t}}\prime \right)=1-\frac{{\text{C}}_{\text{p}}\prime \left(1;\tau \right)}{{\text{C}}_{\text{p}}\prime \left(0;\tau \right)}\\ (\text{b})\frac{\partial {\text{C}}_{\text{p}}\prime \left(\text{x},\tau ,{\text{C}}_{\text{t}},{\text{C}}_{\text{t}}\prime \right)}{\partial \text{x}}=-\frac{\tau }{{\text{V}}_{\text{cap}}}\;\cdot \;{\text{v}}_{\max\_\text{t}}\prime \;.\;\frac{{\text{C}}_{\text{p}}\prime \left(\text{x},\tau ,{\text{C}}_{\text{t}},{\text{C}}_{\text{t}}\prime \right)\;-\;\frac{{\text{C}}_{\text{t}}\prime \left(\tau ,{\text{v}}_{\max\_\text{m}}\right)}{{\text{V}}_{\text{d}}}}{{\text{K}}_{\text{T}}\;+\;{\text{C}}_{\text{p}}\left(\tau ,{\text{C}}_{\text{t}}\right)\;+\;\frac{{\text{C}}_{\text{t}}\left(\tau ,{\text{v}}_{\max\_\text{m}}\right)}{{\text{V}}_{\text{d}}}},\\ \text{ with }{\text{C}}_{\text{p}}\prime \left(0,\tau ,{\text{C}}_{\text{t}},{\text{C}}_{\text{t}}\prime \right)={\text{C}}_{\text{A}}\prime \end{array}\right.$$

for ${{\text{C}}_{\text{p}}}^{\prime }$ and ${{\text{C}}_{\text{t}}}^{\prime }$, for any value of the transit time τ.

In the following, we will not indicate the dependence of ${{\text{C}}_{\text{p}}}^{\prime }$ and ${{\text{C}}_{\text{t}}}^{\prime }$ on v_max_m_ and C_t_, as these functions have been determined previously. We follow the same strategy to solve this system as we did for native glucose. Accordingly, we solve Equation (20)b independently over a grid of values $\left(\tau ,{{\text{C}}_{\text{t}}}^{\prime }\right)$. We compute the corresponding Q′ function on the same grid $\left(\tau ,{{\text{C}}_{\text{t}}}^{\prime }\right)$:

(21) $${\text{Q}}^{\prime }\left(\tau ,{\text{C}}_{{\text{t}}^{\prime }}\right)=1-\frac{{\text{C}}_{{\text{p}}^{\prime }}\left(1;\tau ,{\text{C}}_{{\text{t}}^{\prime }}\right)}{{\text{C}}_{{\text{p}}^{\prime }}\left(0;\tau ,{\text{C}}_{{\text{t}}^{\prime }}\right)}$$

In the second step, we numerically solve the equation:

(22) $$-{\text{v}}_{\max\_\text{m}}\prime \;\cdot \;\frac{\frac{{\text{C}}_{\text{t}}\prime \left(\tau \right)}{{\text{V}}_{\text{d}}}}{{\text{K}}_{\text{M}}+\frac{{\text{C}}_{\text{t}}\prime \left(\tau \right)}{{\text{V}}_{\text{d}}}}+\frac{{\text{C}}_{\text{A}}\prime \;\cdot \;{\text{Q}}^{\prime }\left(\tau ,{\text{C}}_{\text{t}}\prime \right)}{\tau }\;\cdot \;{\text{V}}_{\text{cap}}=0$$

for relevant values of τ, to obtain ${\text{C}}_{\text{t}}^{\prime }\left(\tau \right)$ in steady-state. Having determined the specific value of ${\text{C}}_{\text{t}}^{\prime }\left(\tau ,{\text{v}}_{\max\text{\_}\text{m}}\right)$, we use the previously interpolated Q′ to determine ${\text{C}}^{\prime }\left(\tau ,{{\text{C}}_{\text{t}}}^{\prime }\right)$ for any transit time τ.

From Equation (15), we can determine the mean glucose tracer extraction fraction (GEF′). The LC is then simply derived as the ratio $\text{LC}=\frac{{\text{GEF}}^{\prime }}{\text{GEF}}$.

### Lactate production

We used our model to predict lactate production in different physiological states. To do so, we decompose CMR_glc_ in two parts. CMR_glc_ = M_a_ + M_s_, where M_a_ corresponds to glucose fully oxidized to CO_2_, and M_s_ corresponds to glucose non-oxidatively metabolized to lactate. As each molecule of glucose metabolized non-oxidatively produces two molecules of lactate, the rate at which lactate is produced, P_l_, fulfills:

(23) $${\text{P}}_{\text{l}}=2\;\cdot \;{\text{M}}_{\text{s}}$$

Let us introduce OGI, the ratio between CMRO_2_ and CMR_glc_, with OGI < 6.

(24) $$\left({\text{M}}_{\text{a}}+{\text{M}}_{\text{s}}\right)\;\cdot \text{ OGI}={\text{CMRO}}_{2}$$

As M_a_ corresponds to glucose metabolized oxidatively, *CMRO*_2_/M_a_ = 6.

We can rewrite Equation (24):

(25) $$\left(\frac{{\text{CMRO}}_{2}}{6}+{\text{M}}_{\text{s}}\right)\;\cdot \text{ OGI}={\text{CMRO}}_{2}$$

That is,

(26) $${\text{M}}_{\text{s}}={\text{CMRO}}_{2}\;\cdot \;\frac{6-\text{OGI}}{6\;\cdot \text{ OGI}}$$

Finally,

(27) $${\text{P}}_{\text{l}}={\text{CMRO}}_{2}\;\cdot \;\frac{6-\text{OGI}}{3\;\cdot \text{ OGI}}={\text{CMR}}_{\text{glc}}\;\cdot \;\frac{6-\text{OGI}}{3}$$

with P_l_ being the rate at which lactate is produced, expressed in the same units as CMR_glc_.

## Results

**Figure 2:**
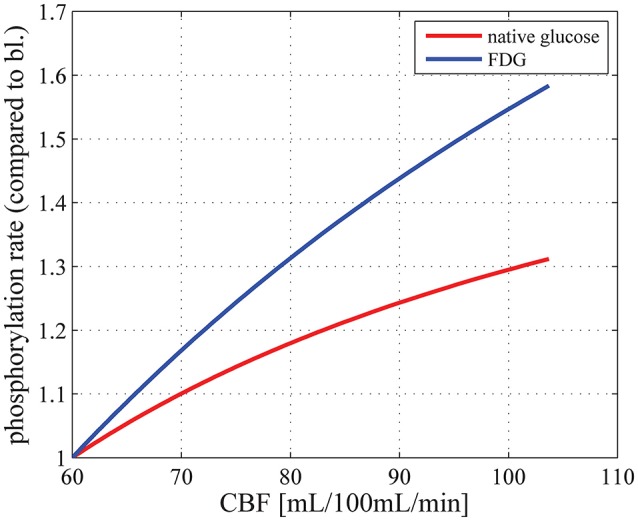
**Relation between CBF and CMR_**glc**_ (red curve) and between CBF and the phosphorylation rate of FDG, that is, CMR_**FDG**_ (blue curve), for physiological states between baseline (MTT = 1.4 ***s***, CTH = 1.33 ***s***) and stimulation (MTT = 0.81 s, CTH = 0.52 s), where CTH is assumed to vary linearly with MTT, using parameter set S_**H**_: v_**max_t**_ = 68 μmol/100 mL/min and K_**T**_ = 50 μmol/100 mL_brain**. CBF is related to MTT through the relation CBF = V_cap_/MTT. CMR_glc_ in baseline condition is equal to 29μmol/100 mL/min. bl, baseline; CBF, cerebral blood flow; V_cap_, cerebral blood volume.

In this section, we first present results regarding the dependency of baseline CMR_glc_ on plasma concentration. We then show how CMR_glc_ is predicted to vary between physiological conditions and compare these changes to those reported in the literature. Experimental evidence suggests that CBF and CMR_glc_ change in proportion (see Paulson et al., 2010, for references and an in-depth discussion). To examine whether our model produces predictions that are consistent with these observations, we determined CMR_glc_ and the phosphorylation rate of FDG (which we refer to as CMR_FDG_) as a function of CBF for (MTT, CTH) states between baseline and activation. Figure 2 shows that an almost linear relation between CBF and CMR_glc,_ as well as between CBF and CMR_FDG_ is obtained, which is consistent with the experimental findings, although increases in CBF are not a prerequisite in the support of increase glucose metabolism (Leithner and Royl, 2014). We also compare changes in CMR_glc_ to that of CMRO_2_, to determine the OGI. Finally, we assess the effects of a change in plasma concentration on the LC and quantify the extent to which it is predicted to change between physiological states.

### CMR_glc_ in baseline condition

**Figure 3:**
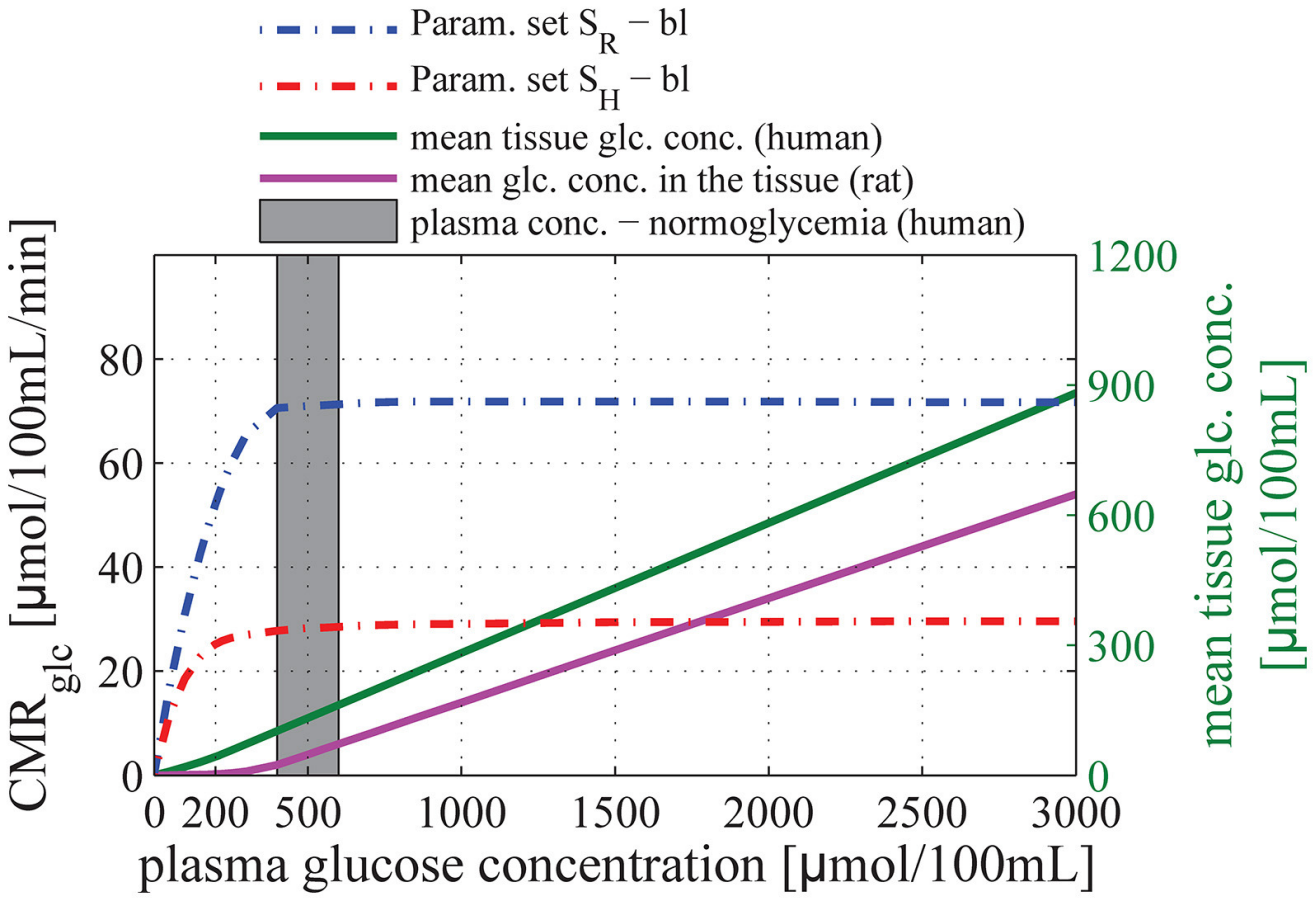
**Relation between glucose concentration in plasma and CMR_**glc**_ in baseline condition**. The purple and green solid lines correspond to the mean glucose concentration in the tissue (input) as a function of arterial glucose concentration according to relations (13) and (14), based on experimental studies by Dienel et al. (1991) and Gruetter et al. (1998), respectively. The blue dotted-dashed line corresponds to predictions obtained using parameter set S_R_: v_max_t_ = 136 μmol/100 mL/min and K_T_ = 150 μmol/100 mL_brain. The red dotted-dashed line corresponds to parameter set S_H_: v_max_t_ = 68 μmol/100 mL/min and K_T_ = 50 μmol/100 mL_brain. The gray area corresponds to conditions of normoglycemia in humans (plasma glucose concentration ranging from 400 to 600 μmol/100 mL).

Figure 3 shows the relation between glucose concentration in plasma and CMR_glc_ in the baseline condition. The CMR_glc_ predicted by our model using parameter set S_R_ (see Methods: Calibration of the Model Parameters) was 72 μmol/100 mL/min, slightly lower than the value reported in conscious rats, see for example (Choi et al., 2002) for review. This underestimation could be caused by the fact that we derived our transport parameters from anesthetized animal data, see discussion in Section Calibration of the Model Parameters. We used a plasma glucose concentration of 1000 μmol/100 mL_plasma in our calculations, as reported for rats under euglycemic conditions. The CMR_glc_ predicted by our model using parameter set S_H_ was 28 μmol/100 mL/min, compared to 30μmol/100 mL/min assumed in human when we derived transport parameters. In this case, we used a plasma glucose concentration of 500μmol/100 mL_plasma, as reported for humans under euglycemic conditions.

For both set of Michaelis-Menten parameters, our model shows that CMR_glc_ in baseline conditions does not depend on arterial glucose concentration until it reaches values as low as 200–300 μmol/100 mL_plasma, concentration at which the mean glucose concentration in the tissue approaches zero. In particular, CMR_glc_ under condition of mild hypoglycemia (C_A_ = 300–400μmol/100 mL_plasma) and hyperglycemia is found to be almost equal to CMR_glc_ under condition of normoglycemia (relative difference lower than 10%).

### Relative changes in CMR_glc_ between physiological states

**Figure 4:**
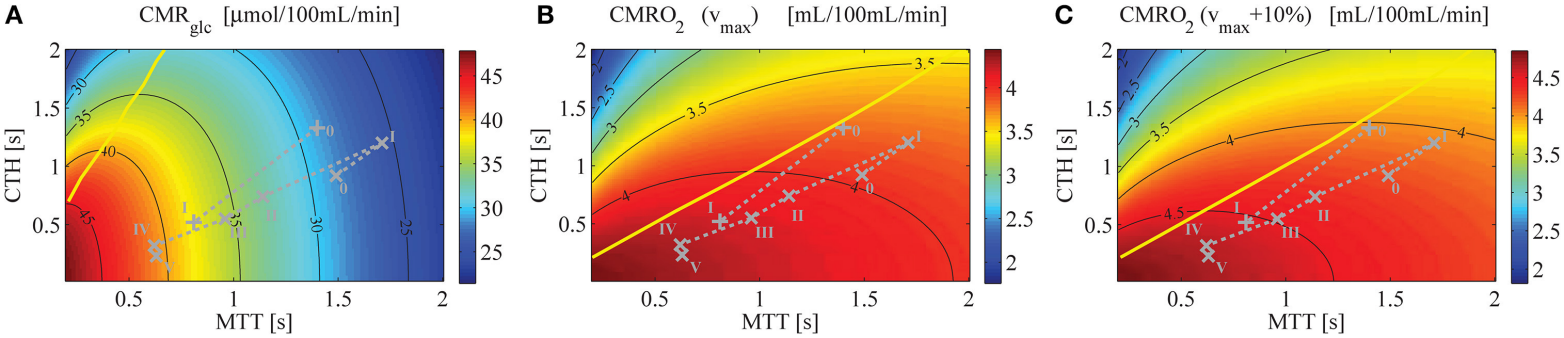
**(A)** CMR_glc_ contour plot, assuming a glucose arterial plasma concentration equal to 500μmol/100 mL, a mean glucose concentration in the tissue under baseline condition equal to 129μmol/100 mL, and Michaelis-Menten parameters for transport of glucose v_max_t_ and K_T_ equal to 68μmol/100 mL/min and 50μmol/100 mL, respectively. **(B)** CMRO_2_ contour plot (Angleys et al., 2015) assuming oxygen metabolism to be governed by Michaelis-Menten kinetics, with parameters K_M_ = 2.71*mmHg* (3.5 μmol/L) and v_max_ = 4.75 mL/100 mL/min. **(C)** CMRO_2_ contour plot assuming v_max_ = 5.23 mL/100 mL/min [i.e., 10% higher than in **(B)]** and making otherwise the same assumptions as in **(B)**. The parameter v_max_ in **(B,C)** is assumed to be constant. The yellow line separates states where a blood flow increase (decrease in MTT) given a fixed CTH will lead to an increased (right side of the line) or decreased (left side of the line) glucose **(A)** or oxygen **(B,C)** consumption, respectively. The roman numeral accompanying each symbol corresponds to physiological data. The numeral “0” stands for resting state, whereas other numerals refer to state of altered basal physiology. Note that the CMR_glc_ and CMRO_2_ iso-contours do not show the same slope for the experimental data used in this figure, indicating that a change in MTT (resp. CTH) will have a strong (resp. moderate) influence on CMR_glc_, and inversely for CMRO_2_. Symbols: +, functional activation (Stefanovic et al., 2008); ×, cortical electrical stimulation (Schulte et al., 2003).

**Figure 5:**
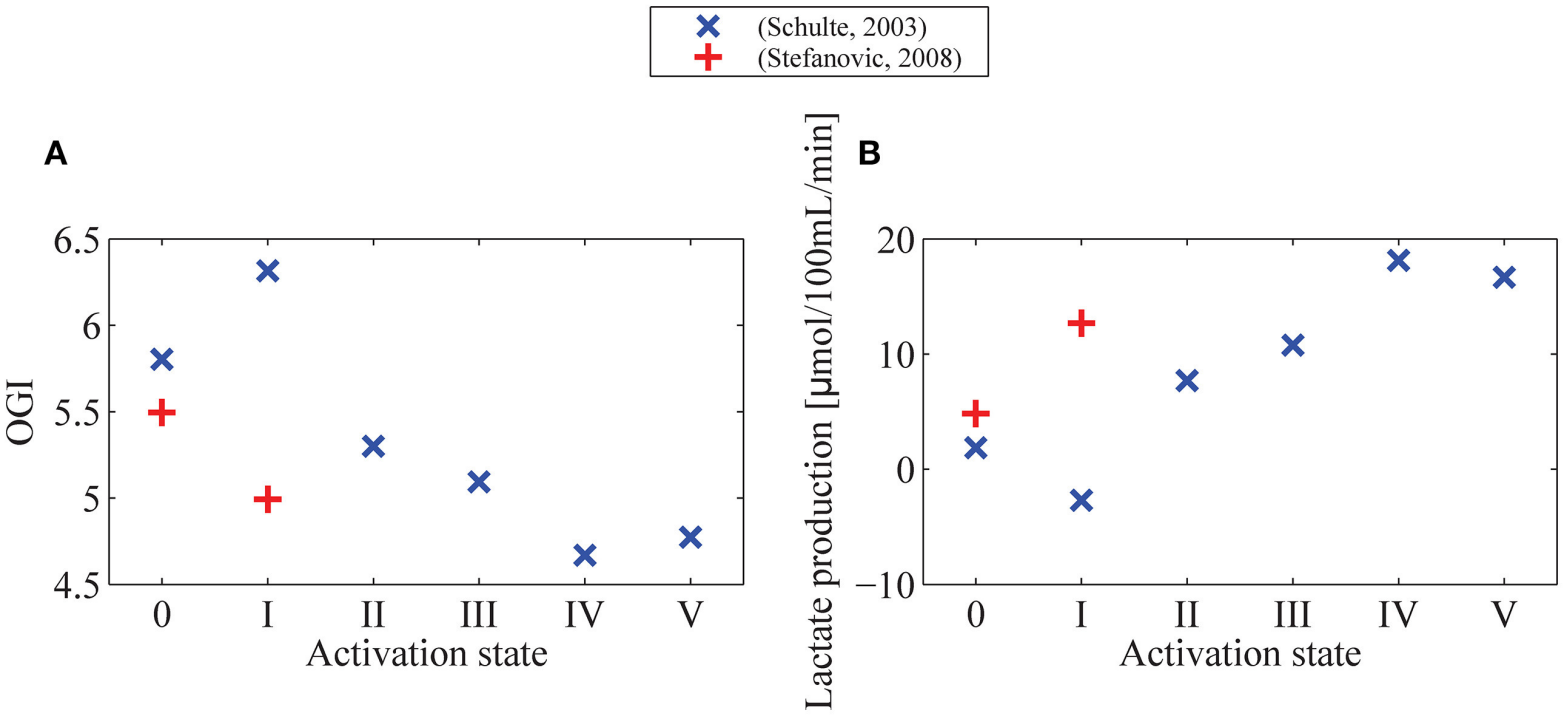
**OGI (A) and lactate production (B) in different physiological conditions indicated by symbols on Figure 4.** We assume a glucose arterial plasma concentration equal to 500 μmol/100 mL, a mean glucose concentration in the tissue under baseline condition equal to 129 μmol/100 mL, and Michaelis-Menten parameters for transport of glucose v_max_t_ and K_T_ equal to 68μmol/100 mL/min and 150μmol/100 mL (Cunningham et al., 1986), respectively. We furthermore assume that oxygen metabolism is governed by Michaelis-Menten kinetics, with parameters K_M_ = 2.71 mmHg (3.5μmol/L) and v_max_ = 4.75 mL/100 mL/min, and that v_max_ increases by 10% from baseline condition to state I (+) or state V (×). In **(B)** lactate production is derived from CMR_glc_ from the formula P_I_ = CMR_glc_·(6−OGI)/3, with P_I_ being lactate production, and OGI being computed in **(A)**. OGI: oxygen-glucose index: CMRO_2_/CMR_glc_.

Figure 4A shows a contour plot of CMR_glc_, using parameter set S_H_, that is, when the model is applied to human. Figures 4B,C show CMRO_2_ contour plots obtained with the model from Angleys et al. (2015) with a maximum metabolic rate for oxygen metabolism (v_max_) equal to 4.75 mL/100 mL/min and 5.23 mL/100 mL/min (that is, 10% higher), respectively. Note that there is no straightforward way to illustrate CMR_glc_ during the transition from the baseline condition (bl) characterized by MTT_bl_, CTH_bl_, and v_max_, to an activated state (act) characterized by MTT_act_, CTH_act_, and v_max_+10% in a single contour plot without specific knowledge on the relation between MTT, CTH, and v_max_. In Figure 5, v_max_ is assumed to increase in proportion to the stimulus intensity, by 10% between baseline and stimulation, which corresponds to the value that yielded the most realistic results in Angleys et al. (2015).

One important observation in Figure 4A is that variations in CMR_glc_ as function of MTT are expected to be higher than variations in CMRO_2_. The OGI ratio therefore depends on the physiological state considered and is expected to decrease during activation, as illustrated in Figure 5A. When we consider changes in MTT and CTH derived from Stefanovic et al. (2008) and Schulte et al. (2003) (Figure 5A), our model predicts that CMR_glc_ increases by 31% (43%) from baseline condition to stimulated state, while CMRO_2_ is expected to increase by only 19% (18%) between baseline and stimulation, resulting in an OGI decreasing by 10–20%, from 5.5 (5.8) to 5.0 (4.7). As a result, lactate production is expected to increase from 3 to 5 μmol/100 mL/min to about 15 to 20μmol/100 mL/min (Figure 5B).

Additional information that can be inferred from Figure 4 is that, for the considered physiological states, CMR_glc_ is influenced primarily by changes in MTT, while a change in CTH has little influence on this variable. This is in contrast to CMRO_2_, which is expected to be influenced primarily by CTH and more moderately by MTT.

Models from Jespersen and Østergaard (2012) and Angleys et al. (2015) predict that for large CTH values and under a condition of fixed CTH, a blood flow increase leads to a decrease in oxygen delivery. This phenomenon which has been referred to as *malignant CTH* is observed in this model with glucose as well. Accordingly, for states on the left hand side of the yellow line in Figure 4A, CMR_glc_ decreases if flow increases under condition of constant CTH.

### LC changes in response to a change in plasma concentration and between physiological conditions

**Figure 6:**
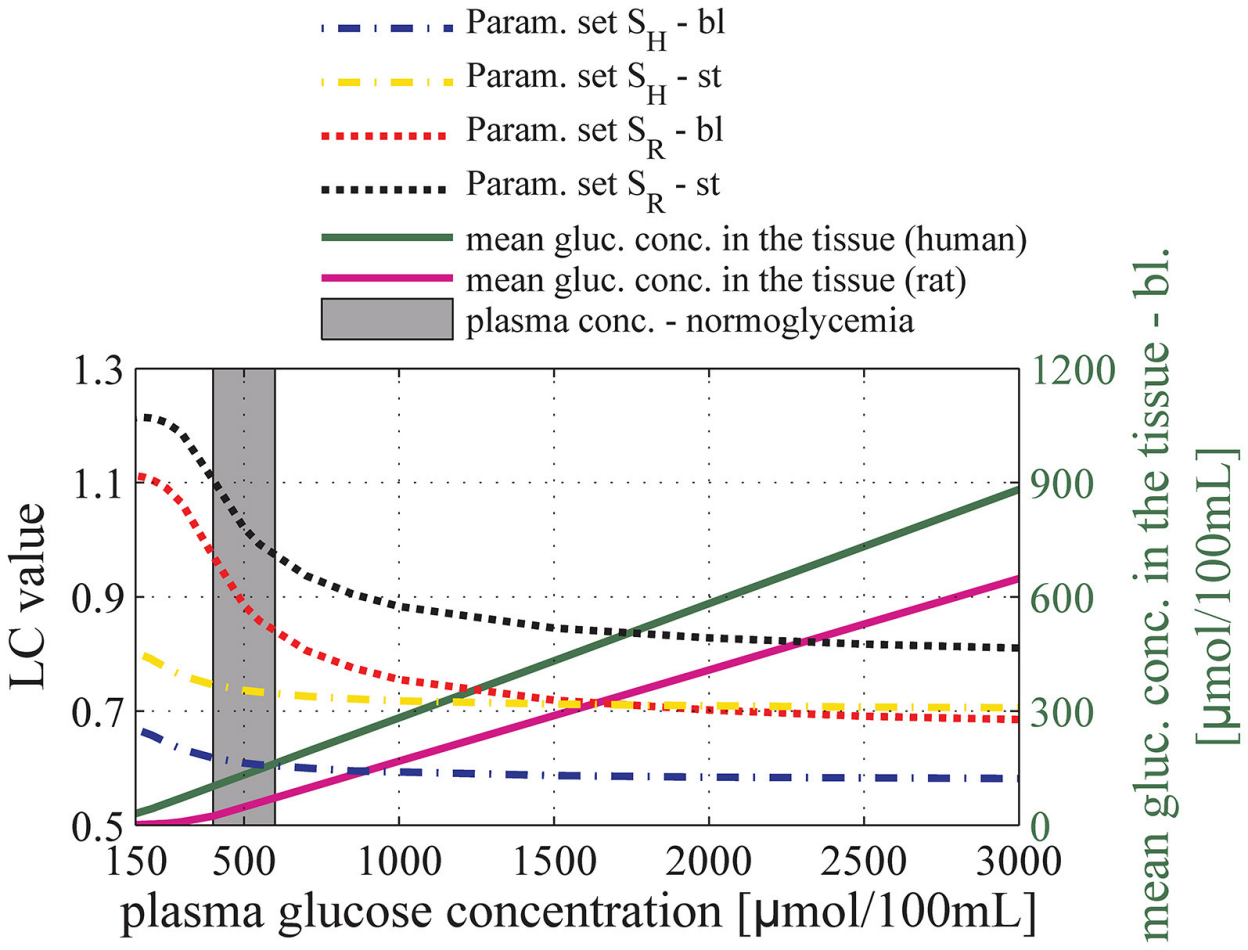
**Relation between glucose concentration in plasma and the lumped constant (LC) for FDG in baseline condition (dashed-dotted line) and during stimulation (dotted line)**. The black and the red lines correspond to predictions obtained using parameter set S_R_: v_max_t_ = 136μmol/100mL/min and K_T_ = 150μmol/100 mL_brain. The yellow and the blue lines correspond to predictions obtained using parameter set S_H_: v_max_t_ = 68 μmol/100 mL/min and K_T_ = 50 μmol/100 mL_brain. The purple and green solid lines correspond to the mean glucose concentration in the tissue (input) as a function of arterial glucose concentration according to relations (13) and (14), based on experimental studies by Dienel et al. (1991) and Gruetter et al. (1998), respectively. The gray area corresponds to conditions of normoglycemia in humans (plasma glucose concentration ranging from 400 to 600μmol/100 mL). bl, baseline condition; st, stimulation.

Figure 6 shows the relation between plasma glucose concentration and the expected value for the LC in baseline condition and during stimulation, assuming the relations in Equations (13) and (14) between ${\bar{\text{C}}}_{\text{t}}^{\text{t}}$ and C_A_. Accordingly, the LC was found to be equal to 0.61 and 0.76 with parameter sets S_H_ and S_R_, respectively under baseline, normoglycemic conditions (C_A_ = 500μ*mol/100 mL* and 1000μmol/100 mL, for set S_H_ and S_R_, respectively). Our model predicts that LC increases under conditions of severe hypoglycemia (C_A_ = 210 μmol/100 mL) to 0.68 (1.11), and decreases under conditions of hyperglycemia to 0.58 (0.69) with parameter set S_H_ (S_R_).

**Figure 7:**
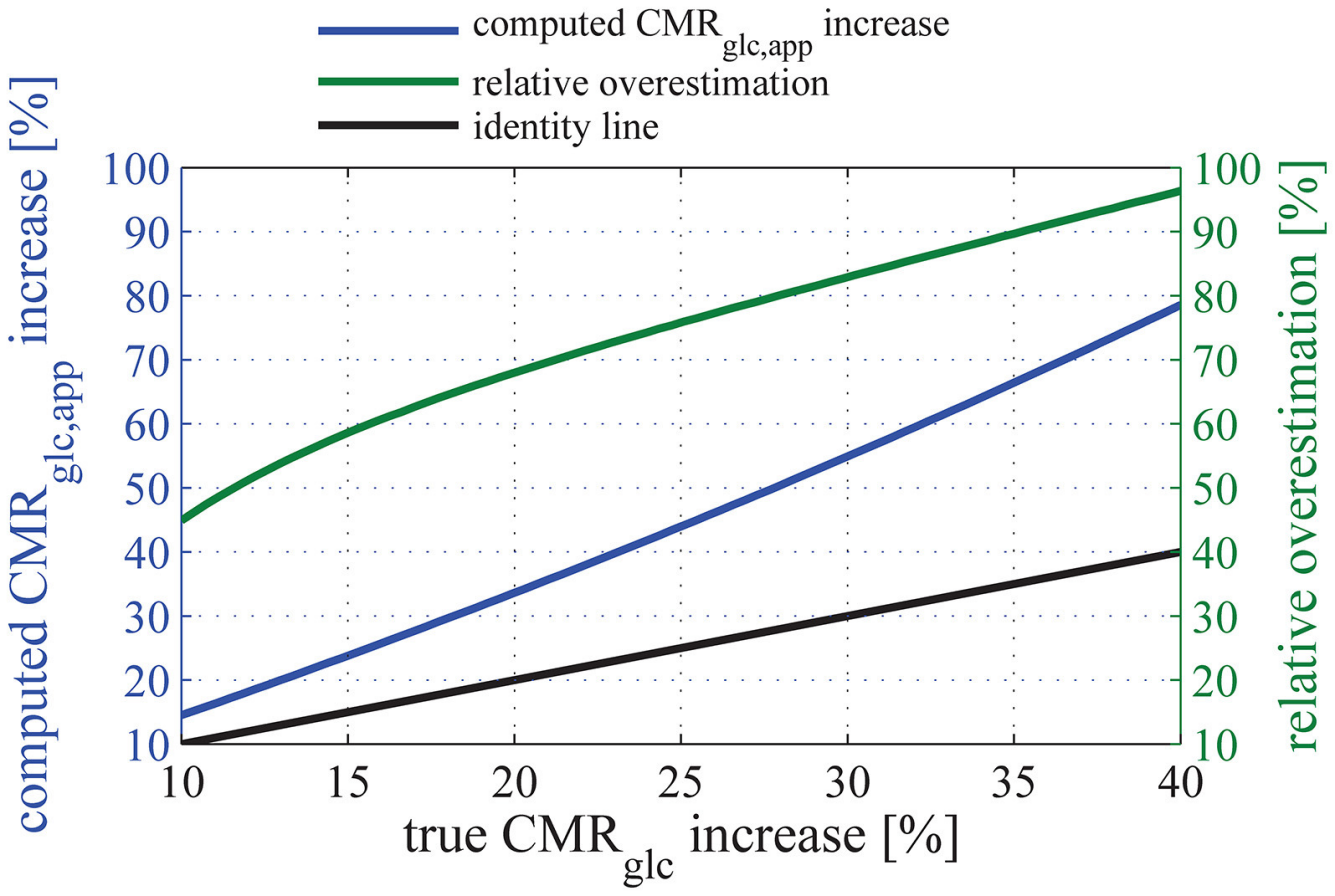
**Relation between the relative (true) CMR_**glc**_ increase, and the apparent relative CMR_**glc, app**_ increase computed when assuming that the lumped constant does not vary between physiologic states (blue line)**. The relationship between CMR_glc, app_, CMR_glc,_ and LC is given in Equation (28). Note that the increase in glucose metabolism is overestimated by more than 50% for any “true” relative CMR_glc_ increases of 12% or more if one neglects the state-dependency of LC. In this plot, parameter set S_H_ is used to describe glucose transport, and we assume that the uptake of glucose tracer from the blood is measured accurately. The green line shows the resulting relative overestimation. The black line is the line of equation y = x.

For arterial glucose concentrations ranging from 400 to 3000 μmol/100 mL, and assuming that changes in MTT and CTH between baseline condition and stimulation are accompanied by a 30% decrease in tissue glucose concentration, our model predicts that LC increases by about 15 and 20% when using Michaelis-Menten parameter sets S_R_ and S_H_, respectively. Figure 7 shows the extent to which these changes in LC would impact the computation of relative increase in CMR_glc_ in human when neglected, provided that the uptake of glucose analog from the blood is measured accurately. In the following, CMR_glc, app_ denotes the apparent metabolic rate of glucose, when neglecting changes in LC value between physiological conditions. Relative change in CMR_glc_, CMR_glc, app_, and LC are related according to the equation:

(28) $$\frac{\Delta {\text{CMR}}_{\text{glc},\text{app}}\left(\text{d}\right)}{{\text{CMR}}_{\text{glc},0,\text{app}}}=\left(1+\frac{\Delta \text{LC}\left(\text{d}\right)}{{\text{LC}}_{0}}\right)\;\cdot \;\frac{\Delta {\text{CMR}}_{\text{glc}}\left(\text{d}\right)}{{\text{CMR}}_{\text{glc},0}}+\frac{\Delta \text{LC}\left(\text{d}\right)}{{\text{LC}}_{0}}$$

It should be noted that Equation (28) predicts CMR_glc, app_ and CMR_glc_ to be equal only when LC remains constant during a change in physiological state (ΔLC = 0). In Equation (28), LC_0_ and CMR_glc, 0_ is the LC and CMR_glc_ value, respectively, in the baseline condition, and ΔLC and ΔCMR_glc_ the subsequent increase in LC and CMR_glc_, respectively. Here, *d* denotes the relative decrease in tissue glucose concentration between conditions. Note that ΔCMR_glc_, ΔLC, and therefore ΔCMR_glc, app_ depend on *d*. The blue curve in Figure 7 shows the relation between CMR_glc_ and CMR_glc, app_, using parameter set S_H_, with *d* varying from 0 to 40%. This range allows ΔCMR_glc, app_ to vary in the range from 7 to 83%, therefore covering the relative increase in glucose metabolism reported in the literature (see Table 3).

**Table 3:** **Comparison between relative increases in glucose metabolism reported in experiments involving NMRS and PET in human**.

**References**	**Technique to measure CMR_glc_ increase**	**Type of stimulation**	**Baseline**	**Relative increase in CMR_glc_ (%)**	**Relative increase in CMR_glc_ after correction (%)**
Chen et al., 1993	NMRS	8 Hz photo stimulation (red-light-emitting-diodes)	NA	22	22
Frahm et al., 1996	NMRS	10 Hz photo stimulation	Darkness	21	21
Fox et al., 1988	PET 18F-DG	Checkboard pattern (10 Hz)	NA	51	28
Phelps et al., 1981	PET 18F-DG	Checkboard pattern (2 Hz)	Eyes closed	27	17
Phelps et al., 1981	PET 18F-DG	More complex scene	Eyes closed	60	32
Vlassenko et al., 2006	PET 18F-DG	Checkboard pattern (8 Hz)	Eyes closed	>50^*^	>28
Villien et al., 2014	PET 18F-DG	Checkboard pattern (8 Hz)	Gray fixation-cross	25	16
Mean				36.6 NMRS: 21.5 PET: 43	23.3 NMRS: 21.5 PET: 24.0
Std/mean (agreement between methods)				0.45	0.27

*Relative increase in the CMR_glc_ after correction refers to CMR_glc_ increase when accounting for the overestimation predicted by our model (Figure 7)*.

*CMR_glc_, cerebral metabolic rate of glucose; NMRS, nuclear magnetic resonance spectroscopy; PET, positron emission tomography*.

* *Estimation based on an experiment where visual stimulation was presented only for the first 5 min of FDG circulation and PET scanning (total duration: 60 min)*.

The function that associates CMR_glc, app_ to CMR_glc_ shows a derivative which is increasing with CMR_glc_. The relative overestimation (green curve in Figure 7) therefore increases as a function of CMR_glc_ and takes particularly high values for high CMR_glc_ relative increases. Because of the non-linearity between relative increases in CMR_glc_ and LC, Equation (28) leads to surprisingly large overestimations of the increase in CMR_glc_ if even small changes in LC are neglected. For example, considering conservative values for LC and CMR_glc_ increases of 10% (see Dienel et al., 1991, for experimental evidence) and 20%, respectively, would lead to an apparent 32% increase in CMR_glc_ (Equation 28), thus overestimating the increase in CMR_glc_ by 60%. Also note that Equation (28) is model-independent and therefore does not rely on simplifying assumptions used in our model. Please refer to the Supplementary Material for details about its derivation.

## Discussion

In this study, we developed a model that takes the effects of tissue glucose concentration and CTH into account when describing glucose extraction in the brain. We employed reversible Michaelis-Menten kinetics, which has previously been shown to support cerebral glucose utilization across a range of arterial glucose/tissue concentration (Choi et al., 2001; van de Ven et al., 2012).

The first main finding in our study is that, similar to oxygen extraction, glucose extraction is not only a function of the CBF and concentration in plasma and tissue, but also depends on capillary transit time heterogeneity. However, as glucose and oxygen transport involve different mechanisms, the changes in CBF and CTH between physiological states do not affect the transport of these two substrates to the same extent. In particular, Figure 4A shows that for physiological CTH/MTT ratio (typically smaller than 2), CMR_glc_ iso-contours are almost vertical, meaning that CTH does not influence glucose uptake to a very large extent. In contrast, for the same CTH/MTT ratio, CMRO_2_ iso-contour slopes are lower (Figures 4B,C), although they would appear to be slightly higher if v_max_ was not kept constant in the figures (see Section Relative Changes in CMRglc between Physiological States for details about this point). Blood flow increases are therefore expected to be less efficient as a means of increasing oxygen than glucose consumption for a given CTH value. As a consequence, glucose delivery can increase more than that of oxygen during enhanced energy demand, favoring non-oxidative glucose consumption. To better illustrate that CTH has a larger influence on oxygen than glucose delivery, we made a simulation in which we assumed that no change in CTH occurred between physiological states, keeping other parameters unchanged. Instead of increasing by 19% (18%) as reported when CTH decreases in parallel with MTT, our model predicts that CMRO_2_ would increase by only 3.6% (7.4%) when using MTT and CTH derived from Stefanovic et al. (2008) and Schulte et al. (2003). In contrast, CMR_glc_ would be less affected, increasing by 23% (38%), compared to 31% (43%) reported earlier in this manuscript. As a consequence, decreases in OGI (16% (22%)) would be larger than predicted when CTH varies (9% (18%)). In other words, during activation, blood flow homogenization is expected to increase oxygen extraction capacity to a greater extent than that of glucose, thus limiting OGI reduction.

The second main finding is that the ratio of glucose tracer to native glucose extraction at steady state (LC) depends on the physiological state we consider, and varies accordingly between baseline and stimulated conditions. This finding is in contrast to previous studies, where this ratio is considered to be constant. We show that neglecting variations in this ratio could lead to overestimations of the relative increase in CMR_glc_ (ΔCMR_glc_/CMR_glc, 0_) between different physiological states of 50% or more. In the following, we discuss how such variations in the LC could reconcile what previously seemed to be incompatible measurements obtained with PET and NMRS, respectively.

### Changes in plasma concentration

Data from the literature reports that glucose metabolism is largely insensitive to changes in plasma concentration. It was therefore important to show that our model is consistent with this data. Accordingly, we tested our model over a range of plasma concentrations to simulate conditions of hypoglycemia and hyperglycemia, keeping the model parameters which describe glucose transport unchanged. We employed two sets of parameters to describe glucose transport for rats and humans, respectively. Our assumption that glucose transport is described by reversible Michaelis-Menten kinetics requires also to assume that tissue glucose concentration varies linearly with plasma concentration in the range from hypoglycemia to hyperglycemia levels (see Equations (13) and (14)), in which CMR_glc_ has been reported to remain almost constant. This linear relation is supported by several experimental studies (Gruetter et al., 1998; Choi et al., 2001; Seaquist et al., 2001; van de Ven et al., 2012). When glucose plasma concentration increases above normoglycemic levels, our model predicts that CMR_glc_ is essentially unchanged. The same observation applies under conditions of hypoglycemia, where the model predicts that CMR_glc_ only decreases slightly until plasma concentrations reaches concentration as low as 200–300 μmol/100 mL_plasma; levels at which glucose concentration in tissue approaches zero. When plasma concentration decreases further, CMR_glc_ is expected to decrease sharply.

Our results are in good agreement with several studies showing that CMR_glc_ only decreases slightly under conditions of moderate hypoglycemia in rats (Bryan et al., 1986). Moreover, several studies report a sharp increase in CBF when glucose plasma concentration decreases to concentration lower than 200μmol/100 mL (Choi et al., 2001), which corresponds to concentration in the tissue close to zero. It suggests that glucose metabolism is more severely impaired when plasma concentration is lower than 200μmol/100 mL_plasma than during mild hypoglycemia, and that CMR_glc_ cannot be maintained at levels observed during normoglycemia. Finally, although CMR_glc_ predicted by our model when applied to rats is slightly lower than reported values (see Section CMR_glc_ in Baseline Condition for explanation of this underestimation), predictions for human were in good agreement with the value we assumed in our calibration.

In summary, our model captures crucial characteristics of glucose delivery to the brain, in particular the remarkable ability of the brain to maintain sufficient glucose metabolism across a wide range of plasma and tissue glucose concentrations. This is in contrast to oxygen metabolism, which is much more sensitive to changes in plasma concentration and CBF. As a result, the brain is much more vulnerable to change in oxygen than in glucose levels (Leithner and Royl, 2014).

### Changes in CMR_glc_ between physiological states

We employed our model to predict CMR_glc_ in different physiological states. These predictions are based on the assumption that the mean glucose concentration in the tissue (i) decreases by 30% between the two physiological states that we took as reference for baseline and stimulation (See sections Calibration of the Models Parameters in Methods and in Discussion for further discussion about this choice), and (ii) is linearly related to MTT. While the first assumption is based on experimental data, a precise relationship between tissue glucose concentration and MTT has yet to be determined, and future work should therefore test the validity of the second assumption. With recent technical developments, tissue glucose concentrations may indeed be measured (Lugo-Morales et al., 2013), and this aspect of our models could therefore be tested experimentally.

Our model predicts that CMR_glc_ increases relatively more than CMRO_2_ between baseline and stimulation. Accordingly, the OGI is predicted to decrease by about 10–20% and lactate production to increase, which is in good agreement with literature reports (Prichard et al., 1991; Madsen et al., 1995, 1998; Frahm et al., 1996). The reasonable number of parameters employed in our model may provide insights into the understanding of the regulation of glucose and oxygen metabolism. Indeed, the strong inhibition of hexokinase in baseline condition allows glucose metabolism to increase more than that of oxygen metabolism, which has been reported to be approximately 80–85% of its maximum value, already at rest (Gjedde et al., 2005). It is also worth noting that the transport capacity of glucose across the BBB varies with the concentration gradient in a non-linear fashion because of glucose transporters properties. In particular, if an increase in concentration gradient is caused by a decrease in tissue glucose levels, as observed during functional activation, glucose transport capacity will increase relatively more than does the gradient. In contrast if the increase in concentration gradient is caused by an increase in arterial glucose concentrations, as observed during normal physiological variations or during hyperglycemia, glucose transport capacity will increase much less than does the gradient. For example, with reversible Michaelis-Menten kinetics (e.g., parameter set S_H_) and the parameters used in our study, a 30% reduction in tissue glucose levels from 132 to 92 μmol/100 mL_brain will increase the concentration gradient by 11% and the net transport rate by 25%. If the same increase in concentration gradient was introduced through an increase in plasma concentration, then the transport rate would increase by only 6.2%. The transport capacity of glucose can thus amplify or attenuate the effect of changes in concentration gradient, and thereby accommodate both high metabolic demands and changes in plasma concentration without the risk of a tissue energy crisis. These uptake properties are reflected by our model response to a change in concentration gradient introduced through a change in plasma concentration and a decrease in tissue concentration, respectively. While a relatively small decrease in tissue concentration is indeed accompanied by a large increase in the net transport rate and hence in CMR_glc_ (Figure 4), a large change in plasma concentration is compensated by a comparatively reduced change in tissue concentration (Figure 3), leading to a stable transport rate and CMR_glc_. These properties are in contrast to free diffusion, which is characterized by a transport rate across the BBB that is proportional to the concentration gradient. These observations further support that non-oxidative glucose consumption may therefore be an inherent consequence of glucose and oxygen extraction, especially during functional activation. Needless to say, the way in which the brains cell types have specialized to utilize the available glucose and lactate as substrates in their metabolism is beyond the scope of this paper.

### Lumped constant

With our model, we can compute the ratio of glucose analog to native glucose extraction (LC). We tested our model over a large range of glucose concentrations, to quantify the extent to which this ratio is predicted to change as a function of the plasma concentration, and to assess whether this is in good agreement with literature reports.

We observe that LC increases from 0.76 with parameter set S_R_ to more than one under severely hypoglycemic conditions, and decreases to 0.68 under hyperglycemic conditions. Variations in LC with parameter set S_H_ are predicted to show the same pattern but with smaller amplitude. These LC values under baseline and normoglycemic conditions are in good agreement with some experiments obtained for FDG in rats (Tokugawa et al., 2007) and in humans (Hasselbalch et al., 2001a), but about 50% higher than others obtained in rats (Huang et al., 1980). See Hasselbalch et al. (2001a) for in-depth discussions of this variability. The variation of the LC under hypo- and hyperglycemic conditions for rats is in line with experimental reports (Suda et al., 1990; Dienel et al., 1991) and modeling (Pardridge et al., 1982; Holden et al., 1991). As pointed out by Pardridge et al. (1982), Crane et al. (1983) and Holden et al. (1991), these variations in LC can be explained by differences between the rate at which native glucose and glucose analog are metabolized and transported across the BBB. Indeed, under hyperglycemia/normoglycemia, glucose metabolism is not limited by transport across the capillary membrane. Under severe hypoglycemia, however, transport capacity limits glucose delivery. As a result, the LC ranges between two extreme values, namely (i) the ratio between the maximal operation rate of hexokinase for glucose analog and native glucose, ${{\text{v}}_{\max\text{\_}\text{m}}}^{\prime }/{\text{v}}_{\max\text{\_}\text{m}}$, equal to 0.3 in our study, which is approached under hyperglycemic conditions, and (ii) the ratio between the maximal rate at which glucose analog and native glucose are transported across the BBB, that is ${{\text{v}}_{\max\text{\_}\text{t}}}^{\prime }/{\text{v}}_{\max\text{\_}\text{t}}$, equal to 1.4, which is approached under hypoglycemic conditions.

In summary, both CMR_glc_ and LC values yielded by our model, as well as their relative changes between two glycemic conditions are consistent with the literature.

We also quantified the change in the value of LC between two physiological states (baseline condition and activation). Our model predicts that, under conditions of normoglycemia and constant blood glucose concentration, the ratio of glucose tracer to native glucose extraction (so-called LC) increases by 15–20% from baseline condition to stimulation, which is in line with earlier predictions by Dienel et al. (1991) based on typical decrease in tissue glucose concentration during activation. As explained in the Supplementary Material and established experimentally (Dienel et al., 1991), we show that this variation is partly due to changes in glucose concentration in the tissue, which does vary substantially from baseline condition to stimulated state (e.g., see Merboldt et al., 1992; Chen et al., 1993; Adachi et al., 1995; Frahm et al., 1996; Mangia et al., 2006; Lin et al., 2012). Accordingly, we believe it is crucial to adjust LC when quantifying the relative increase in CMR_glc_ between two physiological states. Changes in LC between physiological states would have to be determined experimentally (e.g., Dienel et al., 1991), or from model predictions.

### Comparison of relative increases in CMR_glc_ obtained with NMRS and PET

In Table 3, we list several experiments where NMRS and FDG PET were used to quantify relative increases in CMR_glc_ between baseline and an activated state. All experiments involved visual stimulation and all but one (Phelps et al., 1981) used the same kind of stimulus, that is, a checkboard or red diodes flashing at 8 or 10 Hz (see Table 3). The table reveals tendency for PET measurements to yield higher CMR_glc_ increases than those obtained with NMRS (mean relative increase = 43% with PET, 22% with NMRS). When taking into account the change in LC between baseline and stimulation by using the relation between apparent glucose metabolism increase and true glucose increase illustrated in Figure 7, the mean relative increase in CMR_glc_ is reduced to 24% when assessed with PET. The relative standard deviation (standard deviation to mean ratio) is furthermore decreased by 40% when applying the correction, which reflects the better agreement between measures obtained with NMRS and PET after correction than before. Experiments involving NMRS use bigger regions of interest (ROIs) and therefore may contain more white matter than experiments with PET, which might lead to a slight underestimation of the signal during activation. However, in these experiments, ROIs are of comparable sizes and locations, so that signal underestimation with NMRS, if any, is likely to be small. Although more data would be needed to calibrate our model more precisely and hence to achieve a more accurate correction, we believe that such corrections are crucial when inferring glucose uptake from FDG uptake in different physiological states, without which CMR_glc_ estimate may be misleading.

### Applying our model to disease conditions

We applied our model to tumor cells, as an example of possible clinical application. More specifically, we assessed the consequences on the glucose uptake and on the LC of an altered glucose transport or metabolism, such as it has been reported in the literature on tumors: Tumor cells express an isozyme of hexokinase, hexokinase II, which is less susceptible to feedback inhibition by its product, glucose-6-phosphate, than hexokinase in healthy tissue (Bustamante and Pedersen, 1977; Bustamante et al., 1981). When applying our model to tumor cells, to take this reduced inhibition into account, we have increased the maximum rate at which glucose and FDG can be metabolized (v_max_m_ and ${{\text{v}}_{\max\text{\_}\text{m}}}^{\prime }$). We find that the LC increases by 36 and 66%, from 0.61 in baseline condition to 0.83 and 1.01, respectively, under condition of reduced inhibition, while the mean glucose concentration in the tissue is reduced to one half and one fourth of healthy tissue values, respectively. This increase in the LC is accompanied by a 45 and 75% increase in CMR_glc_ compared to healthy tissue. These results are in line with the literature, reporting that most tumor tissues are known to be highly metabolic and depend on aerobic glycolysis (the Warburg effect Warburg, 1956). Spence and colleagues furthermore showed that the LC in tumors was higher than in healthy tissue and was generally found to exceed unity (Spence et al., 1990, 1998).

Our model therefore suggests that smaller inhibition of hexokinase could be a possible factor leading to both increased glucose metabolism and LC value. As discussed in previous sections, underestimation of the LC leads to overestimation of CMR_glc_ as assessed by FDG PET. When neglecting LC increases, quantitative CMR_glc_ estimates in tumors therefore contains little extra information compared to qualitative measures, and could even turn out to be misleading. Overestimating CMR_glc_ in tumors would in turn lead to OGI underestimation and hence to an overestimation of non-oxidative glucose consumption.

Our model also allows us to understand the biophysical mechanisms that lead to a higher LC value. Because hexokinase is less inhibited in tumor than in healthy tissues, glucose phosphorylation is quicker and the equilibrium concentrations in tissues are lower. Consequently, once a glucose molecule crosses the blood-tumor-barrier, its life time in the tissue compartment is reduced and glucose metabolism is limited to a greater extent by glucose transport across the BBB than in healthy tissue. This suggests that glucose transport capacity in tumor cells is reduced relatively to the phosphorylation rate. The LC value therefore becomes weighted to a greater extent by the ratio between native glucose and glucose analog transport rates across the BBB (${{\text{v}}_{\max\text{\_}\text{t}}}^{\prime }/{\text{v}}_{\max\text{\_}\text{t}}$) than by their phosphorylation rates (${{\text{v}}_{\max\text{\_}\text{m}}}^{\prime }/{\text{v}}_{\max\text{\_}\text{m}}$), and hence increases essentially for the same reasons as under conditions of hypoglycemia.

In future work, we could employ our model to better understand disease states related to aerobic glycolysis. For example, in Alzheimer disease (AD), several studies show that areas of the normal human brain with elevated aerobic glycolysis are nearly identical to those that accumulate amyloid and exhibit atrophy and disrupted metabolism in AD (Buckner et al., 2005; Vlassenko et al., 2010). It has therefore been suggested that there might be a link between dependence on aerobic glycolysis and AD. It would be interesting to employ our model to assess the extent to which microvascular dysfunction such as it has been reported in AD and parameters such as OGI are related.

### Calibration of the model parameters

Our model involves several parameters to describe glucose transport. We calibrated most of the parameters with values from the literature. However, the parameters derived in different studies are not easy to compare, because they involve several kinetic models, such as irreversible and reversible Michaelis-Menten kinetics. Choi et al. (2001) made a comparison between parameter values derived when assuming reversible and irreversible Michaelis-Menten, respectively, in human and in rats, while Cunningham et al. (1986) made such as comparison in rats only. Although the parameters of the reversible Michaelis-Menten model take lower values than the non-reversible version, there is no general relation between them. Some studies have included a non-saturable component, often called K_d_, making the comparison between parameters even more difficult. Based on four studies, we applied our model to rats and to humans, using reversible Michaelis-Menten kinetics, in particular to assess how our results depend on a particular choice of parameters. Anesthetics affect the cerebral metabolic rate of glucose (see for example Choi and Gruetter, 2012) and therefore the relationship between glucose concentration in plasma and tissue. While calibrating S_R_, we had to rely on previous studies using reversible Michaelis-Menten kinetics in anesthetized rats (Cunningham et al., 1986; Choi et al., 2001). Choi et al. (2001), however, determined v_max_t_/CMR_glc_ ratio rather than v_max_t_ alone. Provided that anesthesia impacts metabolism rather than glucose transport across the BBB itself, the parameter v_max_t_ is in principle unaffected by the effects of anesthesia. This is in contrast to the second study (Cunningham et al., 1986), where v_max_t_ is determined independently of CMR_glc_. As discussed by Cunningham and colleagues', the value determined for v_max_t_ would have been higher if the experiment had been performed on unanesthetized animals. Our study may therefore have underestimated v_max_t_ in parameter set S_R_, and thus the CMR_glc_ predicted for rats. Note that parameter set S_H_ was based on data from unanesthetized volunteers and the corresponding CMR_glc_ predictions thus apply to awake humans.

Reported values for the Michaelis-Menten constant K_T_ range from −98 to 330 μmol/100 mL in the four studies we used to calibrate our parameters. Moreover, one study (Cunningham et al., 1986) involves rats anesthetized with pentobarbital, which is known to inhibit glucose transport by binding to the glucose transporter itself (el-Barbary et al., 1996; Haspel et al., 1999). The value determined for this parameter (194 μmol/100 mL) is therefore likely to be lower in conscious rats. Accordingly, we chose a slightly lower value for K_T_, 150 μmol/100 mL.

While some of our conclusions are found to be largely insensitive to the choice of parameters, others depend more on the values assigned to describe glucose transport. For example, employing different sets of parameters leads to the same conclusion when one computes CMR_glc_ as a function of arterial plasma concentration, namely that CMR_glc_ is essentially independent to the plasma concentration over a large range of concentration (300–3000 μmol/100 mL) and decreases only when the plasma concentration is so low that glucose concentration in the tissue approaches zero (Figure 3). However, the predicted CMR_glc_ in baseline condition is more dependent on Michaelis-Menten parameters. This is reflected by the results shown in Figure 3, where CMR_glc_ for rats is predicted to be more than two-fold higher than for humans.

In this study, we use our model to assess the extent to which CMR_glc_ and OGI vary between physiological states, and we assume that ${\bar{\text{C}}}_{\text{t}}^{\text{t}}$ decreases by 30% from baseline condition to stimulation. The extent to which tissue concentration decreases during, e.g., photic stimulation has been widely debated and reported values range between 0 and 50% (Collins et al., 1987; Merboldt et al., 1992; Chen et al., 1993; Adachi et al., 1995; Frahm et al., 1996; Mangia et al., 2006; Lin et al., 2012). These differences may be attributed to differences in stimuli involved, experimental protocols and measurement techniques. We estimated our model's sensitivity to this assumption by assuming different ${\bar{\text{C}}}_{\text{t}}^{\text{t}}$ reduction, ranging between 10 and 40%. CMR_glc_ is predicted to increases by 41% (59%), 31% (43%), 22% (29%), and 14% (16%) when ${\bar{\text{C}}}_{\text{t}}^{\text{t}}$ is assumed to decrease by 40, 30, 20, and 10%, and using changes in MTT and CTH derived from Stefanovic et al. (2008) and Schulte et al. (2003). While this large range of predicted CMR_glc_ increases reflects the variability observed in literature reports, it would be valuable to have additional simultaneous recordings of local CMRO_2_, CMR_glc_, CBF, and CTH to sharpen our predictions. We also assume in our model that glucose concentration in plasma and in the tissue are related according to Equations (13) and (14), when the model is applied to rats and humans, respectively. For a given plasma concentration, a large range of tissue concentration have been reported, see for example (Dienel et al., 1991; Madsen et al., 1999) for measures in rats, and therefore other relations could equally have been used. Although our overall conclusions do not depend on these particular choices, more accurate estimates would improve our predictions.

Glucose metabolism is commonly assessed by two different glucose analogs: FDG and 2-DG. FDG is mainly used in humans with PET, while 2-DG is used in rodents with autoradiographic methods. In this study, we focused on the LC in human and chose parameters to describe FDG transport and metabolism rather than 2-DG. While the use of literature values for 2-DG would result in different rate constants, we expect that 2-DG and FDG transport are sufficiently similar for the overall conclusions of our study to hold for both tracers. As discussed above (please see Section Lumped constant in the discussion), the LC can be seen as a weighted average between transport capacity and phosphorylation rate ratios of glucose analogs to native glucose, which in our case are equal to 0.3 and 1.4, respectively. The LC therefore varies between these two values, depending on the physiological condition, which affects the weight given to the transport and to the phosphorylation rates. Consequently, increasing the ratio ${{\text{v}}_{\max\text{\_}\text{t}}}^{\prime }/{\text{v}}_{\max\text{\_}\text{t}}$ to 1.5 as it is sometimes reported for FDG would make LC vary by 22% between baseline condition and stimulation, and by 17% if this ratio is equal to 1.1, compared to 21% in our study, using otherwise the same parameters as in Figure 6, with C_A_ = 500μmol/100 mL and parameter set S_H_.

### Reversible michaelis-menten kinetics

Although modeling of glucose transport across the BBB has been dominated by non-reversible Michaelis-Menten kinetics, we chose to employ reversible Michaelis-Menten kinetics in our study.

Glucose transport across the BBB involves transporters (essentially GLUT-1), which have been studied extensively, and several models have been derived to describe their kinetics (Carruthers and Helgerson, 1991; Baldwin, 1993; Mueckler, 1994; Cloherty et al., 1996). These models are characterized by a high degree of complexity, and all of them allow glucose to bind back to the transporter just after its release, which is supported experimentally (Carruthers and Helgerson, 1991; Cloherty et al., 1996). Although reversible Michaelis-Menten is an oversimplification of these models, it includes this latter possibility, in contrast to non-reversible Michaelis-Menten kinetics, and seems therefore more suitable to describe glucose transport.

Although other studies employ reversible Michaelis-Menten kinetics, only two to our knowledge assessed the relevance of employing reversible instead of non-reversible Michaelis-Menten kinetics (Cunningham et al., 1986; Gruetter et al., 1998). In these studies, it has been shown that employing reversible Michaelis-Menten generally leads to better agreement with experimental data (Cunningham et al., 1986; Gruetter et al., 1998; Seaquist et al., 2001). For example, under constant CMR_glc_, non-reversible Michaelis-Menten-kinetics predicts a non-linear relation between plasma and tissue concentration, the latter being limited by a saturation value. In contrast, reversible Michaelis-Menten predicts a linear relation between tissue and plasma concentration (Gruetter et al., 1998). Experimentally, it has been shown in several studies that concentrations in the tissue and plasma are linearly related to one another over a large range of glucose plasma concentration (250–3000μmol/100 mL) (Gruetter et al., 1998; Choi et al., 2001; Seaquist et al., 2001; van de Ven et al., 2012), suggesting that reversible Michaelis-Menten kinetics is more suitable than non-reversible Michaelis-Menten to describe glucose transport. Finally, reversible Michaelis-Menten kinetics does not involve the use of non-saturable component of unidirectional influx as is the case in several studies employing non-reversible Michaelis-Menten (Cremer and Cunningham, 1979; Pardridge et al., 1982), and therefore better reflects the underlying process involved in facilitated diffusion.

### Neglecting phosphatase activity

In our model, we assume that glucose tracer is trapped in the tissue once it has been phosphorylated, and we therefore neglect the back reaction which is catalyzed by glucose-6-phosphatase, allowing glucose tracer to be dephosphorylated. In studies employing first order rate constants, the constant for this latter reaction is often designated as ${{\text{k}}_{4}}^{*}$ and its value is reported to be equal to approximatively one tenth of ${{\text{k}}_{3}}^{*}$, the first order rate constant for the phosphorylation of glucose by hexokinase. Several studies addressed the effects of phosphatase activity on the estimation of LC and CMR_glc_ (Nelson et al., 1986; Dienel et al., 1988; Schmidt et al., 1992; Gotoh et al., 2000; Hasselbalch et al., 2001b) and suggested that they are negligible for experimental periods shorter than 45 min, and begin to appear with increasing time. Including glucose-phosphatase activity in our model would require the use of additional parameters and to make more assumptions, including assumptions regarding the setup of the experiment (tracer concentration, time of the infusion). While we think that it would be valuable to get quantitatively more precise results, the overall conclusions of our study are not believed to be sensitive to this particular choice.

### Neglecting glucose transfer between extravascular compartments

In our model, we neglect any effects of glucose transfer between extravascular compartments. In reality, glucose might diffuse from nearby microvessels to counteract any effects of CTH, meaning that our model might overestimate tissue concentration-, and therefore concentration gradient heterogeneity. On the basis of CMR_glc_, ${\bar{\text{C}}}_{\text{t}}$, and the diffusion coefficient for glucose in water, the diffusion time of glucose before glycolysis is estimated to be 5 min corresponding to a diffusion distance of 400 μm, which is approximately 10 times that of oxygen, and significantly exceeds the intercapillary distance. This suggests that glucose diffuses not only to the tissue immediately surrounding the nearest capillary, as assumed in our model, but also to the tissue further away. To estimate how this assumption impacts our conclusions, we tested an alternative version of the model, where we assumed no tissue concentration heterogeneity by considering only one extravascular compartment where glucose is well stirred. The results obtained with these two different assumptions lead to almost same results (not shown), suggesting that glucose mixing between extravascular compartments would not affect our conclusion. We also quantified the extent to which CTH introduces heterogeneity at the tissue concentration level, by considering the distribution of extravascular glucose concentrations in the state (MTT_bl_, CTH_bl_) and with the parameter set S_H_. We observed that the highest concentration in the decile (resp. centile) showing the lowest concentration differs by only 22% (resp. 59%) from the lowest concentration in the decile (resp. centile) showing the highest concentration. As a comparison, the transit time of the blood flowing through these compartments differs by 7 (resp. 43)-fold. We speculate therefore that tissue glucose concentration heterogeneity induced by CTH is too small to impact glucose delivery.

## Author contributions

HA developed the biophysical model used in this study, contributed to interpretation of the results and wrote the first draft of the manuscript. SJ developed the study concept, contributed to the development of the biophysical model used in this study and to the interpretation of the results. LØ developed the study concept and contributed to the interpretation of the results.

### Conflict of interest statement

The authors declare that the research was conducted in the absence of any commercial or financial relationships that could be construed as a potential conflict of interest.

## Supplementary material

The model source code can be found online at: https://github.com/HugoAngleys/glucose_extraction

## Abbreviations

ANLS: astrocyte-neuron lactate shuttle
BBB: blood brain barrier
CBF: cerebral blood flow
CMR_glc_: cerebral metabolic rate of glucose
CMRO_2_: cerebral metabolic rate of oxygen
CTH: capillary transit time heterogeneity
GEF: glucose extraction fraction
LC: lumped constant
MTT: mean transit time
NMRS: nuclear magnetic resonance spectroscopy
OEF: oxygen extraction fraction
OGI: oxygen-glucose index
PET: positron emission tomography.
