# Supplementary material for: The Effects of Capillary Transit Time Heterogeneity (CTH) on the Cerebral Uptake of Glucose and Glucose Analogs: Application to FDG and Comparison to Oxygen Uptake
Source: Front Comput Neurosci. 2016 Oct 13;10:103. doi: 10.3389/fncom.2016.00103 (PMC5062759; doi:10.3389/fncom.2016.00103)
Supplement: Supplementary file 1 [file DataSheet1.docx]

Supplementary Material

**The effects of capillary transit time heterogeneity (CTH) on the cerebral uptake of glucose, glucose analogs, and oxygen.**

**Hugo Angleys^1*^, Sune N Jespersen^1,2^, Leif Østergaard^1,3^**

*^1^ Center of Functionally Integrative Neuroscience & MINDLab, Aarhus University, Aarhus, Denmark*

*^2^ Department of Physics and Astronomy, Aarhus University, Aarhus, Denmark*

*^3^ Department of Neuroradiology, Aarhus University Hospital, Aarhus, Denmark*

**^*^Correspondence:**

Hugo Angleys

CFIN, Building 10G, 5th Floor, Nørrebrogade 44

DK-8000 Aarhus C, Denmark

Phone +4578464402

[hugo.angleys@cfin.au.dk](mailto:hugo.angleys@cfin.au.dk)

# Changes in LC assuming no capillary transit time heterogeneity

## Changes in LC under Sokoloff’s assumptions

In this first part, we work under the same assumptions as Sokoloff (Sokoloff et al., 1977) and Gjedde (Gjedde et al., 1981), in order to determine the lumped constant.

Accordingly, we make the assumption that the glucose extraction fraction is small compared to 1 so that the glucose concentration can be considered constant in the capillary (Sokoloff et al., 1977). Glucose and glucose tracer transfer from plasma to the tissue are modelled as a first order process with the rate constant $k_{1}$ and $k_{1}'$ , respectively. Glucose and glucose tracer transfer from the tissue to plasma are modelled as a first order process with the rate constant $k_{2}$ and $k_{2}'$ , respectively. An overview of the model is outlined in Supplementary Figure 1.





**Supplementary Figure 1**: Model overview under Sokoloff’s assumptions. It consists of two compartments, glucose in plasma (concentration C_p_) and in the tissue (concentration C_t_). The glucose metabolism CMR_glc_ depends on the first order rate constant k_3_ and on C_t_. Glucose unidirectional transport between plasma and the tissue is assumed to be a first order exchange process with the rate constant k_1_ and k_2_.

Under these assumptions and at steady state, the net flux of glucose and glucose tracer from plasma equal glucose metabolism:

$$\left\{\begin{matrix} k_{1}C_{p}-k_{2}\frac{C_{t}}{V_{d}}=k_{3}\frac{C_{t}}{V_{d}} \\ k_{1}'C_{p}'-k_{2}'\frac{C_{t}'}{V_{d}'}=k_{3}'\frac{C_{t}'}{V_{d}'} \end{matrix}\right.$$

The lumped constant $L$, which by definition is equal to the glucose tracer to the native glucose extraction fraction ratio can be written as

$$\frac{k_{3}'C_{t}'/C_{p}'\cdot V_{d}'}{k_{3}C_{t}/C_{p}\cdot V_{d}}$$

From equation , this ratio is equal to:

$$L=\frac{k_{3}'\cdot k_{1}'\cdot\left(k_{3}+k_{2}\right)}{k_{3}\cdot k_{1}\cdot\left(k_{3}'+k_{2}'\right)}$$

The expression of the lumped constant in eq. is similar to that given by Gjedde (eq. 25 in (Gjedde et al., 1981)) and Sokoloff (Sokoloff et al., 1977), taking in the latter case $V_{m}'\cdot K_{m}/V_{m}\cdot K_{m}'$instead of $k_{3}'/k_{3}$, where V_m_, K_m_, V’_m_ and K_m_’ refer to the Michaelis-Menten parameters of hexokinase, which catalyzes the reaction where glucose is transferred from the tissue to the metabolic pool.

## Changes in LC assuming that glucose transport between plasma and the tissue is governed by Michaelis-Menten kinetics.

In this second part, we assume that glucose transport between plasma and the tissue is governed by reversible Michaelis-Menten kinetics, making otherwise the same assumptions as in the previous section. An overview of the model is outlined in Supplementary Figure 2.





**Supplementary Figure 2**: Model overview assuming that glucose transport between plasma and tissue is governed by Michaelis-Menten kinetics. It consists of two compartments, glucose in plasma (concentration C_p_) and in the tissue (concentration C_t_). The glucose metabolism CMR_glc_ depends on the first order rate constant k_3_ and on C_t_

Considering reversible Michaelis-Menten kinetics, the unidirectional flux from the tissue to plasma can be written as:

$$v_{-}'=v_{\max\_t}'\cdot\frac{\frac{C_{t}'}{V_{d}'}}{K_{T}'\cdot\left(1+\frac{C_{p}}{K_{T}}+\frac{C_{t}}{V_{d}.K_{T}}\right)+\frac{C_{t}'}{V_{d}'}+C_{p}'}$$

If $C_{t}'\ll C_{t}$, equation can be written simplified:

$$v_{-}'=v_{\max\_t}'\cdot\frac{\frac{C_{t}'}{V_{d}'}}{K_{T}'\cdot\left(1+\frac{C_{p}}{K_{T}}+\frac{C_{t}}{V_{d}\cdot K_{T}}\right)}$$

If we consider native glucose, we can write in a similar fashion:

$$v_{-}=v_{\max\_t}\cdot\frac{\frac{C_{t}}{V_{d}}}{K_{T}\cdot\left(1+\frac{C_{t}'}{V_{d}'\cdot K_{T}'}+\frac{C_{p}'}{K_{T}'}\right)+\frac{C_{t}}{V_{d}}+C_{p}}$$

We can simplify equation under the assumption that C_t_’<<C_t_, to

$$v_{-}=v_{\max\_t}\cdot\frac{\frac{C_{t}}{V_{d}}}{K_{T}+\frac{C_{t}}{V_{d}}+C_{p}}$$

Note the C_t_-dependence in these expressions, especially considering that C_t_, K_T_’ and K_T_ are of the same order of magnitude.

Employing similar expression for the unidirectional transfer of glucose from tissue to plasma, the net flux of glucose through the BBB can be expressed as:

$$v_{+}-v_{-}=v_{\max\_t}\cdot\frac{C_{p}-\frac{C_{t}}{V_{d}}}{K_{T}+C_{P}+\frac{C_{t}}{V_{d}}}$$

If we consider steady state, this flux will equal the flux of glucose metabolism:

$$v_{+}-v_{-}=v_{\max\_t}\cdot\frac{C_{p}-\frac{C_{t}}{V_{d}}}{K_{T}+C_{p}+\frac{C_{t}}{V_{d}}}=k_{3}\cdot\frac{C_{t}}{V_{d}}$$

For the tracer, we can write (see section II.4):

$$v_{+}'-v_{-}'=v_{\max\_t}'\cdot\frac{C_{p}'-\frac{C_{t}'}{V_{d}'}}{K_{T}'+\frac{K_{T}'}{K_{T}}C_{P}+\frac{K_{T}'}{K_{T}}\cdot\frac{C_{t}}{V_{d}}}=k_{3}'\cdot\frac{C_{t}'}{V_{d}'}$$

We can derive the lumped constant $L$ as:

$$L=\frac{\frac{k_{3}'C_{t}'}{C_{p}'\cdot V_{d}'}}{\frac{k_{3}\cdot C_{t}}{C_{p}\cdot V_{d}}}=\frac{v_{\max\_t}'}{v_{\max\_t}}\cdot\frac{K_{T}\cdot\left(1+\frac{C_{p}}{K_{T}}+\frac{C_{t}}{V_{d}\cdot K_{T}}\right)+v_{\max\_t}\cdot V_{d}/k_{3}}{K_{T}'\cdot\left(1+\frac{C_{P}}{K_{T}}+\frac{C_{t}}{V_{d}\cdot K_{T}}\right)+v_{\max\_t}'\cdot V_{d}'/k_{3}'}$$

$$L=\frac{v_{\max\_t}'}{v_{\max\_t}}\cdot\frac{\frac{\alpha}{\beta}+1}{\frac{K_{T}'}{K_{T}}\cdot\frac{\alpha}{\beta}+\frac{k_{3}\cdot V_{d}'\cdot v_{\max\_t}'}{k_{3}'\cdot V_{d}\cdot v_{\max\_t}}}$$

with $\alpha=K_{T}+C_{p}+C_{T}/V_{d}$ and $\beta=v_{\max\_t}\cdot V_{d}/k_{3}$

For the sake of simplicity, let us set, as a preliminary approach $K_{T}=K_{T}'$, $V_{d}=V_{d}'$

Numerical application with $\frac{v_{\max\_t}'}{v_{\max\_t}}=1.4$ and $\frac{k_{3}'}{k_{3}}=0.3\text{ }$

$$L=1.4\cdot\frac{\alpha/\beta+1}{\alpha/\beta+4.67}$$

Assuming that glucose concentration C_t_ decreases from $132\text{ }\mu\text{mol/100mL}$ to $92\text{ }\mu\text{mol/100mL}$ between baseline and stimulation according to equation (14) in the main text, with $C_{A}=500\text{ }\mu\text{mol/100mL\_plasma}$, V_d_ = 0.77, using values of parameter set S_H_ ($v_{\max\_t}=68\text{ }\mu mol/100mL\_brain/\min$ and $K_{T}=50\text{ }\mu mol/100mL\_brain$), and assuming that CMR_glc_ increases from $28\text{ }\mu\text{mol/100mL/min}$ to $37\text{ }\mu\text{mol/100mL/min}$ between physiological states (as predicted by our model when using parameter set S_H_), $\alpha/\beta$ is predicted to vary from 2.95 to 5.17, which corresponds to a lumped constant equal to 0.73 and 0.88 (i.e. 21% increase) at baseline and during stimulation, respectively.

Using values of parameter set S_R_ ($v_{\max\_t}=136\text{ }\mu mol/100mL\_brain/\min$and $K_{T}=150\text{ }\mu\text{mol/100mL\_brain}$), and assuming that glucose concentration C_t_ decreases from $168\text{ }\mu\text{mol/100mL}$ to $118\text{ }\mu\text{mol/100mL}$ between baseline and stimulation according to equation (13), with C_A_ = 1000 μmol/100mL_plasma, and that CMR_glc_ increases from $71\text{ }\mu\text{mol/100mL/min}$ to $86\text{ }\mu\text{mol/100mL/min}$between baseline condition and stimulation (as predicted by our model when using parameter set S_R_), $\alpha/\beta$ is predicted to vary from 5.59 to 9.11, which corresponds to a lumped constant equal to 0.90 and 1.03 (i.e. 14% increase) at baseline and during stimulation, respectively.

These values of the lumped constant are to be compared to those obtained with our model in Supplementary Figure 6. In particular, changes in the lumped constant between baseline condition and activation predicted by this ‘simplified’ version of our model are close to the 21% and 17% increase in the lumped constant value predicted by our model when using parameter sets S_H_ and S_R_, respectively. This simplified version, which does not take CTH into account, therefore provides some understanding into the mechanisms leading to a change in the lumped constant.

# Derivation of $\tilde{h}$ from the capillary transit time h :

To compute the mean glucose concentration in the tissue compartment, we sum the contribution of the function C_t_ for every capillary weighted by the distribution which gives the volume fraction of capillaries with a given transit time, as opposed to the fraction of the flow (implicit for the capillary transit time distribution h).

By definition of h, the blood flow with a transit time between $\tau$ and $\tau+d\tau$ can be written $CBF\cdot h\left(\tau\right)\cdot d\tau$. Noting that this quantity equals the ratio of the capillary volume with a transit time between $\tau$ and $\tau+d\tau$ to the transit time $\tau$ leads to the equation

$$CBF\cdot h\left(\tau\right)\cdot d\tau=\tilde{h}\left(\tau\right)\cdot d\tau\cdot V_{cap}/\tau$$

that is

$$h\left(\tau\right)\cdot\tau/MTT=\tilde{h}\left(\tau\right)$$

where we use the relation $MTT=CBF/V_{cap}$. Hence, if h is a gamma distribution with parameters$\left(\alpha,\beta\right)$, $\tilde{h}$ is a gamma distribution with parameters $\left(\tilde{\alpha},\tilde{\beta}\right)=\left(\alpha+1,\beta\right)$

# Derivation of equation (28)

Glucose metabolism $M_{glc}$ and net phosphorylation rate of a tracer (FDG in that example) $M_{FDG}$ are related through the equation

$$M_{FDG}=M_{glc}\cdot L$$

where L is the true value of the lumped constant. Now if one works with an approximation of the lumped constant $\tilde{L}$, the apparent metabolism of glucose $M_{glc,app}$and $M_{FDG}$are related through the following equation

$$M_{FDG}=M_{glc,app}\cdot\tilde{L}$$

From equations and we have:

$$M_{glc,app}\cdot\tilde{L}=M_{glc}\cdot L$$

If we assume that the lumped constant value has been determined successfully in baseline condition ($\tilde{\text{L}}_{0}=L_{0}$) and that its variation between physiological states are neglected ($\tilde{\text{L}}_{1}=L_{0}$), then:

$$\left\{\begin{matrix} M_{glc,app,0}=M_{glc,0} \\ M_{glc,app,1}=M_{glc,1}\cdot\frac{L_{1}}{L_{0}} \end{matrix}\right.$$

And the relative variation in glucose can be written as

$$\frac{M_{glc,app,1}-M_{glc,app,0}}{M_{glc,app,0}}=\frac{M_{glc,1}L_{1}/L_{0}-M_{glc,0}}{M_{glc,0}}$$

That can be rewritten as

$$\frac{M_{glc,app,1}-M_{glc,app,0}}{M_{glc,app,0}}=\frac{M_{glc,1}-M_{glc,0}}{M_{glc,0}}\cdot\left(1+\frac{L_{1}-L_{0}}{L_{0}}\right)+\frac{L_{1}-L_{0}}{L_{0}}$$

**References:**

Gjedde, A., Hansen, A.J., Quistorff, B., 1981. Blood-Brain Glucose Transfer in Spreading Depression. J. Neurochem. 37, 807–812. doi:10.1111/j.1471-4159.1981.tb04465.x

Sokoloff, L., Reivich, M., Kennedy, C., Rosiers, M.H.D., Patlak, C.S., Pettigrew, K.D., Sakurada, O., Shinohara, M., 1977. The [14c]deoxyglucose Method for the Measurement of Local Cerebral Glucose Utilization: Theory, Procedure, and Normal Values in the Conscious and Anesthetized Albino Rat1. J. Neurochem. 28, 897–916. doi:10.1111/j.1471-4159.1977.tb10649.x
